# The Seed Germination Test as a Valuable Tool for the Short-Term Phytotoxicity Screening of Water-Soluble Polyamidoamines

**DOI:** 10.3390/polym16121744

**Published:** 2024-06-19

**Authors:** Elisabetta Ranucci, Sofia Treccani, Paolo Ferruti, Jenny Alongi

**Affiliations:** Dipartimento di Chimica, Università degli Studi di Milano, Via C. Golgi 19, 20133 Milano, Italy; elisabetta.ranucci@unimi.it (E.R.); sofia.treccani@unimi.it (S.T.); paolo.ferruti@unimi.it (P.F.)

**Keywords:** seed germination test, polyamidoamine, flame retardant, phytotoxicity, eco-toxicity

## Abstract

Six differently charged amphoteric polyamidoamines, synthesized by the polyaddition of *N*,*N*′-methylenebisacrylamide to alanine, leucine, serine, arginine (M-ARG), glutamic acid (M-GLU) and a glycine/cystine mixture, were screened for their short-term phytotoxicity using a seed germination test. *Lepidium sativum* L. seeds were incubated in polyamidoamine water solutions with concentrations ranging from 0.156 to 2.5 mg mL^−1^ at 25 ± 1 °C for 120 h. The seed germination percentage (*SG*%), an indicator of acute toxicity, and both root and shoot elongation, related to plant maturation, were the considered endpoints. The germination index (*GI*) was calculated as the product of relative seed germination times relative radical growth. The *SG*% values were in all cases comparable to those obtained in water, indicating no detectable acute phytotoxicity of the polyamidoamines. In the short term, the predominantly positively charged M-ARG proved to be phytotoxic at all concentrations (*GI* < 0.8), whereas the predominantly negatively charged M-GLU proved to be biostimulating at intermediate concentrations (*GI* > 1) and slightly inhibitory at 2.5 mg mL^−1^ (0.8 < *GI* < 1). Overall, polyamidoamine phytotoxicity could be correlated to charge distribution, demonstrating the potential of the test for predicting and interpreting the eco-toxicological behavior of water-soluble polyelectrolytes.

## 1. Introduction

Water-soluble polymers are widely used in various consumer product formulations, e.g., personal care products, detergents, pharmaceutic and paint formulations [1], and agricultural fertilizers [2]. Cationic polyelectrolytes are also largely used as flocculants in the biological wastewater treatment of sludge [3,4]. After usage, water-soluble polymers are normally disposed of in landfills, incinerated, or directly dumped into rivers and aquatic environments. Because of their solubility in water, they are filtered and transported to aquifers, and now they are ubiquitous contaminants. The water-soluble polymers present in aquatic environments may alter the physico-chemical properties of the aqueous matrix [5]. For instance, their interaction with persistent organic pollutants can increase the bioavailability of the latter and facilitate their entry into the food chain [6]. In the case of polyelectrolytes, the presence of charged groups can cause their aggregation with organic matter [7] and minerals [8], forming floating composites that can eventually sink. They can cause toxic effects related to their charge density and positive/negative charge ratio [9]. Notwithstanding their ubiquity, the danger of water-soluble polymers, particularly the charged ones, is often underestimated, as demonstrated by the fact that, to the best of our knowledge, they have not been included yet in European circular economy action plans. However, their REACH (Regulation, Evaluation, Authorization, and Restriction of Chemicals; UE 1907/2006) registration is under discussion [9,10]. For all the above reasons, the levels of water-soluble polymers in waters must be monitored and robust tests adopted to assess their eco-toxicity [11].

Several protocols for the short-term toxicity screening of hazardous chemicals have been proposed by international agencies for the protection of the environment [12,13,14]. These tests can demonstrate whether contaminants are bioavailable. In fact, the presence of a contaminant does not indicate an adverse effect, since it can only have toxic effects if it is bioavailable [15]. The seed germination test, a quick and reliable test that is routinely used for assessing the quality of compost for agricultural use [16,17,18], has proven to be a useful tool for evaluating the phytotoxicity of several contaminants in different environments. These include, for instance, heavy metal ions [19,20,21,22] and drugs [23,24] in polluted water and/or soils, hydrocarbons in contaminated soils [25], herbicides [26], and microplastics [27,28,29,30]. The effect on seed germination of bio-based biodegradable and petroleum-derived non-biodegradable polymers has been investigated [16].

**Scheme 1:**
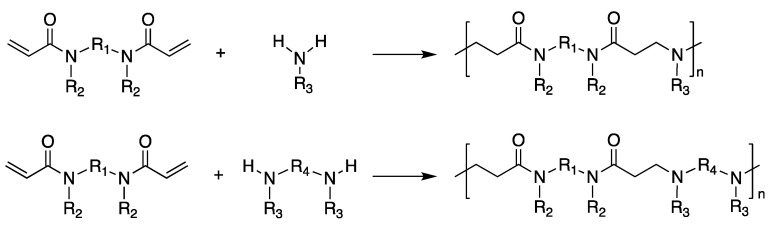
General synthesis of linear polyamidoamines.

Polyamidoamines (PAAs) are synthetic polymers endowed with peculiar structural properties [31]. PAAs are obtained by the aza-Michael stepwise polyaddition of prim- or bis-sec-amines to bisacrylamides (Scheme 1).

Their synthetic processes are green and easily scalable, since they are performed in water at room temperature, with no added catalysts and producing no byproducts. Nearly all conceivable bisacrylamides and prim- or sec-amines can be used as monomers to introduce different organic functions as pendants [32]. PAAs can be designed to be biocompatible. All of them slowly degrade in water at pH ≥ 7 [33,34]. They are normally cationic due to the presence of tert-amine groups in the main chain. However, PAAs carrying acid pendants are amphoteric [32].

In a recent study [35], the seed germination test was adopted to evaluate the phytotoxicity of a water-soluble amphoteric glycine-derived polyamidoamine, named M-GLY, which is currently being investigated as a flame retardant for cotton [36,37]. M-GLY water solutions with concentrations from 0.156 to 5.0 mg mL^−1^ were tested, together with solutions of M-GLY oligomers containing 20–30% acrylamide terminals, which are potentially more toxic than high molar mass polymers at the same concentrations. M-GLY did not inhibit the germination of *Lepidium sativum* L. seeds (watercress) or seedling elongation, as revealed by the values of the germination index (*GI*), defined as the product of the relative radical growth times the relative seed germination, which was invariably higher than 1 (Equation (1)). Acrylamide-terminated M-GLY oligomers inhibited root elongation only at the maximum tested concentration, that is, at 5.0 mg mL^−1^.

(1) $$GI=\frac{Number\;of\;germinated\;seeds\;in\;test\;sample}{Number\;of\;germinated\;seeds\;in\;water}\times \frac{Average\;radicle\;length\;in\;test\;sample}{Average\;radicle\;length\;in\;water}$$

In this study, the effectiveness and robustness of the seed germination test for assessing the phytotoxicity of water-soluble polymers has been investigated using *Lepidium sativum* L. seeds as a viable model and applying the test to six amphoteric α-amino acid-derived PAAs, which are currently being investigated as flame retardants for cotton. Several parameters have been considered to assess the response of *Lepidium sativum* L. seeds exposed to PAA water solutions of different concentrations [18,38], including their germination percentage, root and shoot elongation, and germination index. Additionally, the effect of PAAs on the early stages of germination has been monitored. A large set of raw data and an accurate statistical analysis allowed us to critically analyze the different test parameters and demonstrate their versatility and usefulness in evaluating the phytotoxicity of water-soluble polymers.

## 2. Materials and Methods

### 2.1. Materials

*N*,*N*′-methylenebisacrylamide (99%), alanine (99%), leucine (99%), serine (99%), arginine (99%), glutamic acid (98%), glycine (98%), cystine (99%), lithium hydroxide monohydrate (98%), and 1 mol L^−1^ hydrochloric acid were supplied by Sigma-Aldrich (Milan, Italy) and used as received. *Lepidium sativum* L. (watercress) seeds, a top-quality line, were purchased from Germisem Sementes (Oliveira do Hospital, Portugal) and stored in a dry location. Only seeds less than 6 months old were tested, and we discarded those that showed imperfections upon visual analysis. The average seed length and width, measured using a Dino-Lite Edge digital microscope AM7115MZT model with 5 MP resolution (VWR International s.r.l., Milano, Italy), were 2.7 ± 0.2 mm and 1.4 ± 0.2 mm, respectively. Deionized water (18 MΩ cm, pH 6.5) purified with a Q20 Millipore Milli-Q^®^ apparatus system (Sigma-Aldrich, Milan, Italy) was used in the experiments. The pH of all tested solutions and controls was checked with a Metrohm 826^®^ pH-meter (Metrohm Italiana s.rl., Origgio, Varese) at the start and at the end of the experiment.

### 2.2. Synthesis of Polyamidoamines (PAAs)

Synthesis of M-ALA: *N*,*N*′-methylenebisacrylamide, alanine, and lithium hydroxide monohydrate were dispersed in deionized water inside a plastic vial. The reaction mixture was heated to 50 °C for 5 days in the dark under nitrogen. After this time, it was diluted to 25 mL with deionized water, the pH was adjusted to 4.5 with 37% HCl, and finally the mixture was ultrafiltered using an Amicon^®^ system equipped with a regenerated cellulose membrane with a 3000 g mol^−1^ molar mass cut-off (Sigma-Aldrich, Milan, Italy). The product was retrieved by freeze-drying the retained portion.

All other PAAs were synthesized following the same procedure adopted for M-ALA, using the conditions shown in Table 1.

### 2.3. Seed Germination Test

Seed germination tests were performed using *Lepidium sativum* L. seeds in accordance with the US Environmental Protection Agency (EPA) protocol [12,43]. PAA solutions with concentrations of 0.156, 0.313, 0.625, 1.25, and 2.5 mg mL^−1^ were prepared by diluting aliquots of a 2.5 mg mL^−1^ stock solution prepared by dissolving 250 mg PAA in 100 mL deionized water and adjusting the pH to 7.0 for all PAAs, except for M-GLY_50_-CYSS_50_, whose solutions were conditioned at pH 7.5 due to the solubility limitations of these polymers. This concentration range was selected considering the EPA guidelines, which suggest at least 6 different concentrations increasing in a geometric series where the ratio is between 1.5 and 2.0 [43].

Seeds exposed to deionized water and to 0.1% K_2_Cr_2_O_7_ served as the negative and positive controls, respectively. The test procedure involved evenly distributing 10 seeds on an 85 mm diameter filter paper disk inside a 90 mm diameter polystyrene Petri dish and then soaking them in 4 mL of the test solution (or water). Experiments were carried out in the dark: the Petri dish was closed with Parafilm M^®^ and stored in a black plastic bag at 25 ± 1 °C. A quadruplicate test was performed for each condition (4 × 10 seeds per dish) and, in parallel, a quadruplicate test was conducted with the seeds incubated in deionized water. After 120 h, the germinated seeds were counted and seedling, shoot, and root lengths were measured using a Dino-Lite Edge digital microscope AM7115MZT model with 5 MP resolution (VWR International s.r.l., Milano, Italy) equipped with DinoXcope software (1.5.47). The results of the seed germination test were reported as seed germination percentage (*SG*%), relative seed germination (*RSG*), relative radicle growth (*RRG*), and germination index (*GI*), obtained using Equations (2)–(5) [16]:

(2) $$SG\%=\frac{Number\;of\;germinated\;seeds}{Number\;of\;tested\;seeds}\times 100$$

(3) $$RSG=\frac{Number\;of\;germinated\;seeds\;exposed\;to\;test\;sample\;}{Number\;of\;germinated\;seeds\;in\;water}$$

(4) $$RRG=\frac{Average\;radicle\;length\;in\;test\;sample}{Average\;radicle\;length\;in\;water}$$

(5) GI=RSG×RRG

### 2.4. Seed Germination Kinetics

A couple of *Lepidium sativum* L. seeds were incubated in a 2.5 mg mL^−1^ PAA test solution (0.5 mL) inside a quartz cuvette and visually inspected over 24 h in sunlight using a Dino-Lite Edge digital microscope AM7115MZT model with 5 MP resolution (VWR International s.r.l., Milano, Italy), operating at 50X magnification. All tests were started at the same time of day. A couple of *Lepidium sativum* L. seeds exposed to deionized water (0.5 mL) inside a quartz cuvette were visually analyzed in parallel. All tests were carried out in triplicate. Video recording and processing were performed using the DinoXcope software. The times for maximum imbibition, rupture of the testa tegument, and seed germination, identified as a protrusion of 2 mm radicle, were measured.

### 2.5. Data Analysis

#### 2.5.1. Outlier Data

Outlier data include experimental values that deviate significantly from most values within a given dataset [44]. To identify the outliers within each length measurement dataset, the following steps were performed:

1. For each series, the measurements collected in each experiment were sorted in ascending order.
2. Measurements were then divided into quartiles (Q_1_–Q_4_), that is, four intervals containing approximately the same number of data points.
3. The Q_1_, Q_2_, Q_3_, and Q_4_ values were identified as the highest values in each group.
4. Outliers were identified as the values falling outside the following range:

[Q_1_ − *k* (Q_3_ − Q_1_); Q_3_ + *k* (Q_3_ − Q_1_)]

where Q_1_ and Q_3_ represent the first and third quartiles and *k* is a non-negative constant indicating the width of the range, beyond which a value is considered abnormal. For this study, *k* was set to 1 [45].

Figure S1 in the Supplementary Materials reports an example of a data distribution set relative to the seedling length values obtained by exposing seeds to deionized water, with an indication of the outliers.

#### 2.5.2. Experimental Error

For each dataset, the average length was calculated by excluding outliers, and the experimental error was quantified using the confidence function, as defined in Equation (6):

(6) $$Confidence=\left(1-\alpha \right)\frac{\sigma }{\sqrt{n}}$$

where α is the significance level 0.05, which represents a 95% confidence level according to the EPA guidelines [12]; σ is the population’s standard deviation; and *n* is the sample size.

#### 2.5.3. *T*-Test

In this study, the *t*-test [44,45] was used as a statistical model to compare the response of *Lepidium sativum* L. seeds exposed to each PAA concentration with the response of seeds exposed in parallel to the negative control. Specifically, it was used to evaluate whether there was a significant difference between the means of two independent groups, namely, the lengths of seeds exposed to a PAA water solution and to the negative control, respectively. Here, a null hypothesis (H_0_) was assumed when no significant difference in the means of the two groups was observed; the alternative hypothesis (H_1_) was assumed in the presence of a significant difference.

The *t*-test was performed according to the following steps:

1. The test of variances was performed to define the most suitable equation to determine the t value [44]. In the present study, different variances of measurement were calculated for group 1 (seeds exposed to PAA) and 2 (negative control).
2. The unequal variances *t*-test, also called the Welch’s *t*-test, was used for calculating the *t*-statistic (*t*) value, following Equation (7):

(7) $$t=\frac{{\bar{x}}_{1}-{\bar{x}}_{2}}{\sqrt{\frac{\sigma_{1}^{2}}{n_{1}}+\frac{\sigma_{2}^{2}}{n_{2}}}}$$

where ${\bar{x}}_{1}$ and ${\bar{x}}_{2}$ are the average values, *σ*^2^_1_ and *σ*^2^_2_ are the variances, and *n*_1_ and *n*_2_ are the sizes of groups 1 and 2, respectively.

3. Then, the degrees of freedom (*df*) were calculated following Equation (8):

(8) $$df=\frac{(\frac{\sigma_{1}^{2}}{n_{1}}+{\frac{\sigma_{2}^{2}}{n_{2}})}^{2}}{\frac{{\left(\frac{\sigma_{1}^{2}}{n_{1}}\right)}^{2}}{n_{1}-1}+{\frac{{\left(\frac{\sigma_{2}^{2}}{n_{2}}\right)}^{2}}{n_{2}-1}}^{\;}}$$

where *σ*^2^_1_ and *σ*^2^_2_ are the variances and *n*_1_ and *n*_2_ are the sizes of groups 1 and 2, respectively.

4. Finally, the *p*-value was calculated as the two-tailed probability of the *t*-distribution and compared to a chosen significance level of 0.05. If the *p*-value was less than the significance level, the null hypothesis H_0_ was rejected, indicating a significant difference in the mean values of groups 1 and 2.

## 3. Results and Discussion

### 3.1. Water-Soluble Amphoteric Polyamidoamines: Their Synthesis and Ionic Species Distribution

**Scheme 2:**
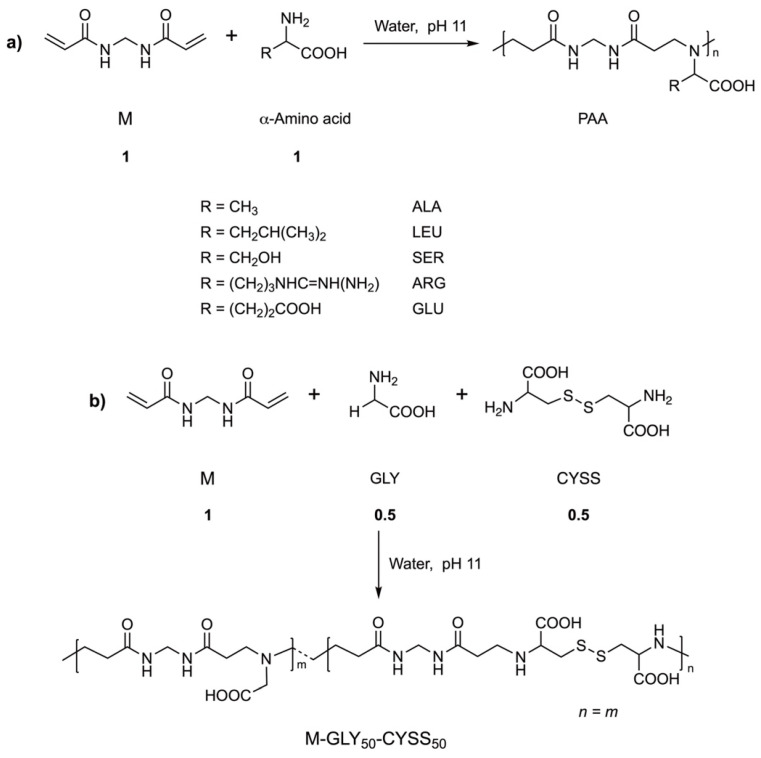
Synthesis of PAA homopolymers (**a**) and of the glycine–cystine-derived PAA copolymer (**b**). For the sake of simplicity, charges have been omitted.

**Figure 1:**
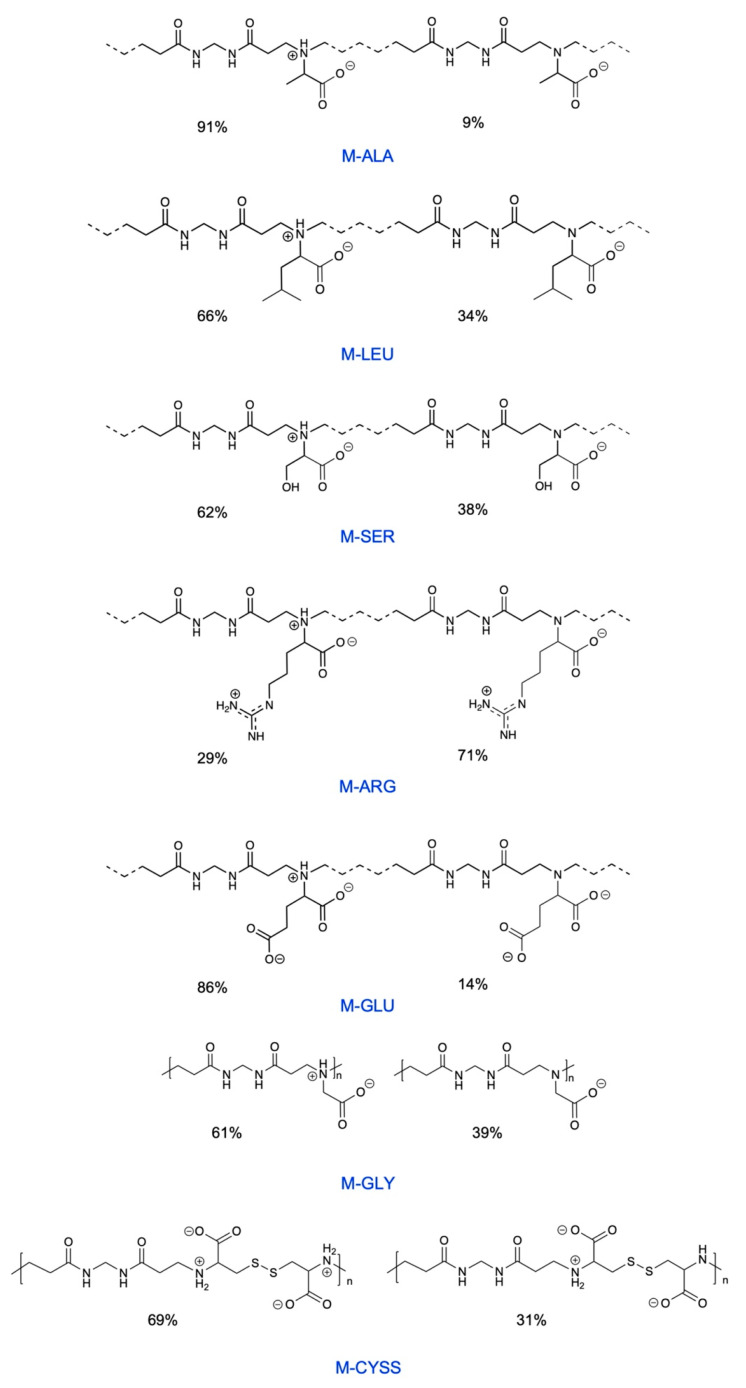
Ionic species distribution of α-amino acid-derived PAAs at pH 7.0, except for M-GLY_50_-CYSS_50_, for which the pH considered is 7.5. In the case of the M-GLY_50_-CYSS_50_ copolymer, the repeating units M-GLY and M-CYSS are considered separately. The PAA codes are specified in Table 1.

The objective of this study was to demonstrate the robustness and versatility of the seed germination test in evaluating the eco-friendliness of water-soluble amphoteric polymers, a category of polymers that is currently attracting much attention due to the damage they can exert on the environment, potentially comparable to that of microplastics [6]. To this end, six water-soluble polyamidoamines (PAAs) derived from α-amino acids, which have been shown to act as flame retardants (FRs) for cotton, were investigated. These PAAs, labelled M-ALA, M-LEU, M-SER, M-ARG, M-GLU, and M-GLY_50_-CYSS_50_ (Figure 1 and Table 2), contain carboxyl- and amine groups in their repeat units. Therefore, they are amphoteric polyelectrolytes that present well-defined pH-dependent ionic species distributions. One of the research objectives was indeed to correlate the test results to the charge density and the ratio between positive and negative charges in the PAA repeat units. PAA samples were synthesized according to a classical procedure, by the aza-Michael polyaddition of *N*,*N*′-methylenebisacrylamide to α-amino acids, that is, alanine, leucine, serine, arginine, and glutamic acid (Scheme 2a), in a 1:1 mole ratio and with a glycine/cystine mixture in a 1:0.5:0.5 molar ratio, respectively (Scheme 2b). Their synthesis was carried out in a pH 11 water solution at a 50 wt.% solid concentration and for different reaction times, due to the different reactivities of α-amino acids caused by the different steric hindrances of the side substituents [31,46]. The raw products were retrieved by freeze-drying with no further purification. The chemical structures of the final products were confirmed by ^1^H-NMR spectroscopy (Figures S2–S7, respectively, in the Supplementary Material). Their average molar masses were calculated from the ^1^H-NMR spectra, from the ratio between the integrals of the resonance peaks relative to the internal units and the integrals of the resonance peaks relative to the terminal repeat units. The spectra were consistent with molar masses higher than 10,000 g mol^−1^ for all PAAs, except for M-ALA and M-SER, whose molar masses were 4000 g mol^−1^.

The ionic species distributions shown in Figure 1 were determined from the speciation curves shown in Figures S8–S13, obtained from the *pKa* values of the ionizable functions present in them. The titration curve of M-GLU was determined following the approach described in the Supplementary Materials. The calculated isoelectric points (IPs, see Table 2) are indicative of an overall negative charge in their repeat units at pH 7.0 (pH 7.5 in the case of M-GLY_50_-CYSS_50_) and indicative of an overall negative charge, except for M-ARG, which is predominantly cationic. M-ALA is weakly anionic and features 9% repeat units bearing a single net negative charge, with the remaining being zwitterionic (net average charge per repeat unit −0.09; positive/negative charge ratio 0.91). M-LEU, M-SER, and M-GLY_50_-CYSS_50_ are moderately anionic and feature 34%, 38%, and 35% repeat units bearing a single net negative charge, respectively, with the remaining being zwitterionic (net average charge per repeat unit −0.33, −0.38, and −0.35; positive/negative charge ratio 0.66, 0.62, and 0.72, respectively). M-GLU is highly anionic and features 14% repeat units with two negative charges and the remaining percentage with two negative charges and one positive charge (net average charge per repeat unit −1.14; positive/negative charge ratio 0.43). M-ARG features 29% repeat units with two positive charges, while the are remaining zwitterionic (net average charge per repeat unit +0.29, positive/negative charge ratio 1.29).

The acid–base properties of the reported PAAs can be compared to those of linear PAAs with different structures, whose *pK_a_* vary in a wide range and whose ionic species distribution vary from prevailingly positive to negative [47].

### 3.2. Effect of PAAs on the Early Stages of Seed Germination

**Figure 2:**
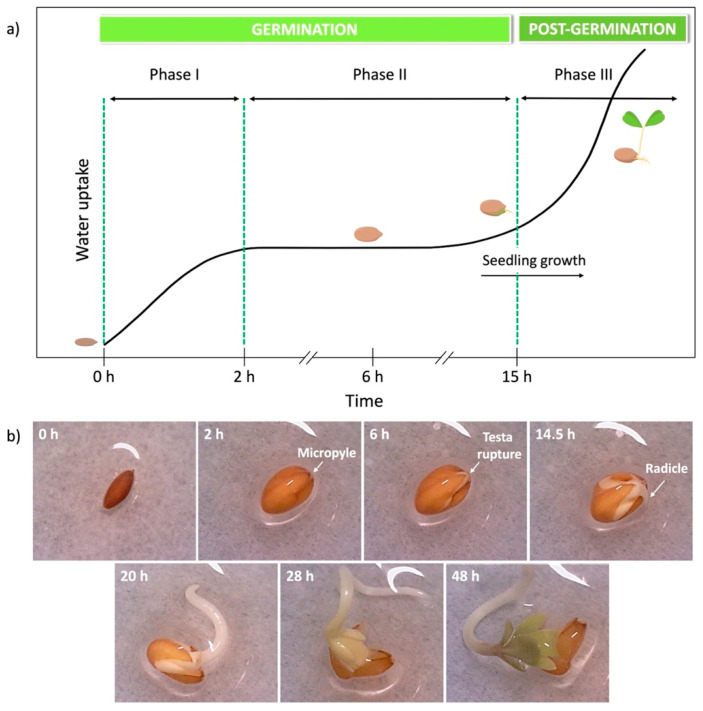
Water uptake during the phases of seed germination (**a**). Morphology of *Lepidium sativum* L. seeds at different stages of the germination process (**b**).

Before performing germination tests, the kinetics of the early stages of the germination of *Lepidium sativum* L. seeds was studied to ascertain whether further endpoints could be identified. Seed germination is a complex process which involves several biochemical, physiological, and morphological transformations within the seed [48]. It starts with the absorption of water through the micropyles, small pores present on the testa tegument, by the dormant dry seeds and is completed when the radicle protrudes from the covering structures by 2 mm. The progress of seed germination is strictly related to its water uptake rate and can be subdivided into three phases (Figure 2) [49]. During phase I (the early germination phase or imbibition), seeds are rapidly imbibed by water, causing the seed coat to expand and soften until it reaches the early plateau of water uptake. Moisture triggers an increase in cellular respiration and activates hormone activity (Figure 2a).

Phase II (the interim or lag phase) coincides with the water uptake plateau, during which hydration is minimal, and it ends with radicle protrusion through the seed covering layers. This phase is characterized by increased metabolic and cellular activity, which generate reserve mobilization and fermentation. The activation of enzymes is partly from the reactivation of stored enzymes and partly from the synthesis of these enzymes during the germination initiation process.

For a seed to complete germination, the growth potential of the radicle must overcome the tissue resistance of the seed covering layers. For many species, including *Lepidium sativum* L., testa rupture and endosperm rupture are two sequential germination steps [50]. The emergence of the radicle is the first visible symptom of germination, and it results from the elongation of cells rather than from cell division.

Phase III (the later germination phase, also called the post-germination phase), corresponds to seedling development. In the germination of *Lepidium sativum* L. seeds, as in all dicotyledonous seeds, the roots grow out from the seed coat first, whereas, in the germination of monocotyledonous seeds, the coleorhiza is the first part to grow out of the seed coat [51].

**Figure 3:**
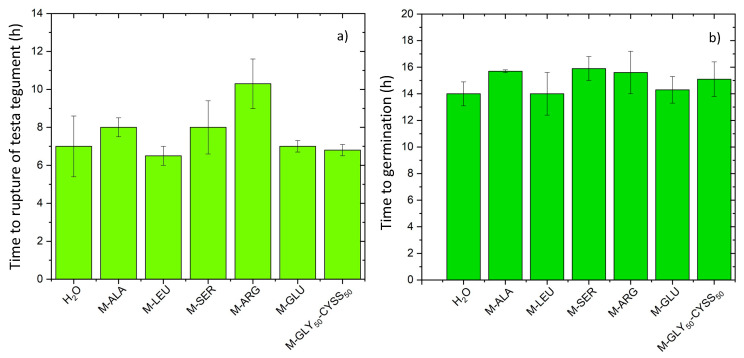
Germination kinetics of *Lepidium sativum* L. seeds exposed to 2.5 mg mL^−1^ PAA solutions for 24 h in sunlight at 25 ± 1 °C (**a**). Time to rupture of testa tegument (**b**). Time to seed germination, identified as the protrusion of a 2 mm radicle. The time to maximum imbibition was 2 h ± 0.5 in all cases. The collected values are reported as the average of three measurements plus their standard deviation.

The early phases of the germination of *Lepidium sativum* L. seeds in water and in PAA water solutions were monitored by an optical microscopic analysis (Figure 2b) to highlight the possible effects of the interference of PAAs on the biochemical processes that take place during embryo (seed) maturation. The times for complete imbibition, the rupture of the testa tegument (preliminary to radicle protrusion), and germination, corresponding to 2 mm radicle emergence, were detected. The maximum tested concentration of the PAA solutions was 2.5 mg mL^−1^ in the seed germination tests (Section 3.3). The time for complete imbibition was, in both water and PAA solutions, approximately 2 h. Interestingly, in all experiments, the germination time did not significantly differ from that detected in water (Figure 3b). However, the time to rupture of the testa tegument significantly differed from that detected in water in the experiment carried out with M-ARG (Figure 3a), the only predominantly cationic PAA. This result suggests the interference of M-ARG in the biochemical processes that occur during embryo maturation; indeed, this PAA proved to be the most phytotoxic of all, as will be shown in the following sections. It may therefore be preliminarily concluded that the analysis of the initial stages of germination could be a useful bioindicator and, additionally, that the early phases of the germination process of *Lepidium sativum* L. seeds proceed smoothly in PAA solutions in the selected conditions.

### 3.3. Effect of PAAs on Seed Germination, Root, and Shoot Elongation of Lepidium sativum *L.*

The seed germination test provides multiple levels of information, the first of which is the assessment of acute toxicity through the identification of the EC_50_ value, defined as the chemical concentration that reduces the number of seeds germinated within the test period by 50% compared to the negative control. The indirect sublethal effects of the water-soluble substances absorbed by the seedlings are normally estimated by monitoring the effect of their exposure on root elongation [52].

**Figure 4:**
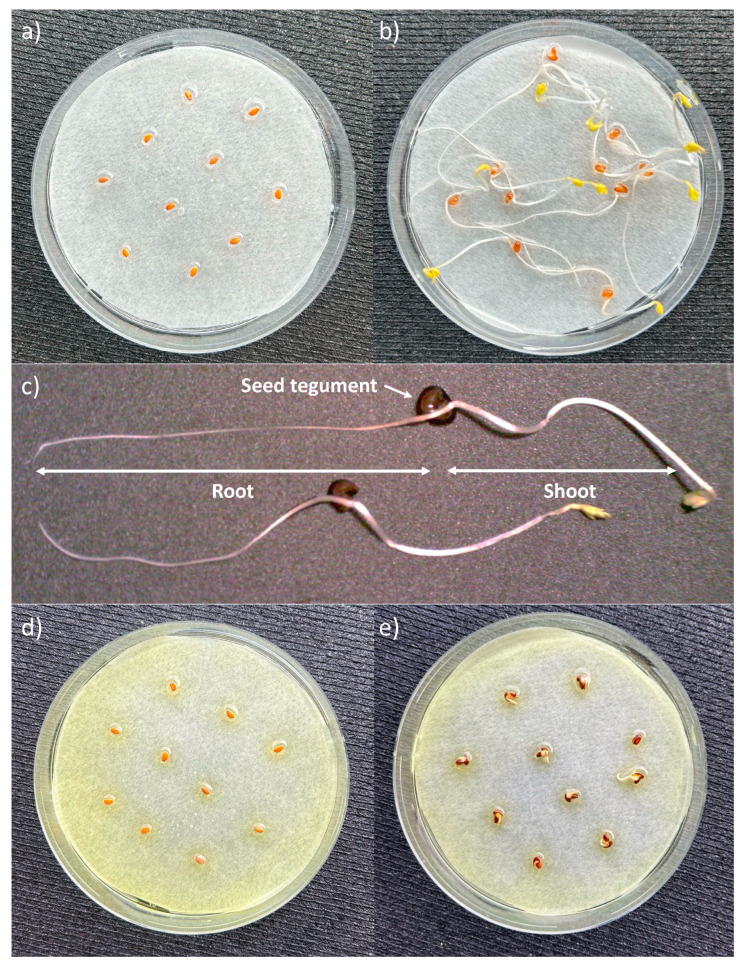
*Lepidium sativum* L. seedling growth after seed exposure to deionized water at 0 (**a**) and 120 h (**b**). Optical microscope image of seedlings grown in deionized water (**c**). Effect of seed exposure to the positive control (0.1% K_2_Cr_2_O_7_) at 0 h (**d**) and 120 h (**e**).

The experimental protocol used in this study was developed based on the requirements of the US Environmental Protection Agency (EPA) in terms of the general experimental conditions, including the type of water, temperature, duration, darkness, and concentration range [12,43]. *Lepidium sativum* L. seeds were chosen as a viable model due to their rapid and easy germination. As stated in Section 2, *Lepidium sativum* L. seeds were exposed to PAA water solutions with concentrations of 0.156, 0.313, 0.625, 1.25, and 2.5 mg L^−1^ for 120 h, in the dark, at 25 °C and pH 7.0, except in the case of M-GLY_50_-MCYSS_50_, for which the tests were carried out at pH 7.5. Notably, the maximum tested value was higher than the 1 mg L^−1^ threshold recommended by the reference protocol [43]. The philosophy was to stress the test to assess its versatility. For each polymer concentration, experiments were performed in quadruplicate, and a quadruplicate test was carried out with seeds incubated in water. Therefore, a total of 1200 seeds were exposed to distilled water. The great number of seeds tested allowed us to perform a reliable statistical analysis of the collected data. Following the EPA guidelines [15], results were considered unacceptable when the average germination of the negative control was less than 90%, and the test was repeated. The average values of the parameters collected for the negative control were SG% = 98 ± 2%, total seedling length = 12 ± 2 cm, shoot length = 3.2 ± 0.4 cm, and radicle length = 8 ± 2 cm. Figure 4 shows the aspect of seeds at the 0 time point and at 120 h in a single Petri dish in the case of the negative (Figure 4a,b) and positive controls (Figure 4d,e).

**Figure 5:**
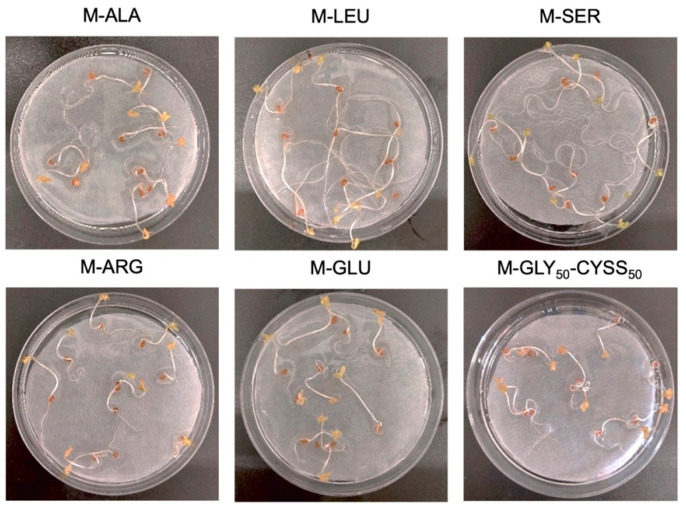
*Lepidium sativum* L. seedling growth after seed exposure to 2.5 mg mL^−1^ PAA solutions for 120 h.

Figure S14 shows a set of 10 *Lepidium sativum* L. seedlings obtained by exposing seeds to deionized water as a negative control. A more detailed picture of seedlings grown in the same experiments is also given in Figure 4c, indicating the shoot and the radicle shape and the relative length observed in that experiment. Figure 5 shows the germination and seedling growth when *Lepidium sativum* L. seeds were exposed to 2.5 mg mL^−1^ PAA solutions for 120 h. The pictures, relative to the experiments conducted with 0.156, 0.313, 0.625, and 1.25 mg mL^−1^ PAA solutions, are shown in Figures S15–S18.

**Figure 6:**
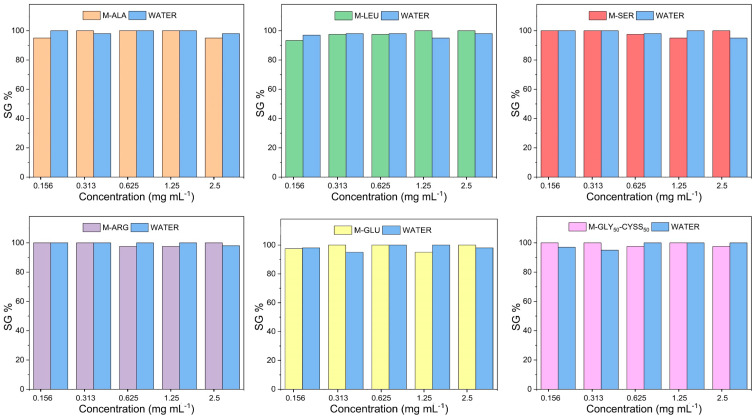
Concentration dependence of the seed germination percentage, SG%, of *Lepidium sativum* L. seeds exposed to PAA solutions for 120 h (colored bars). For each PAA concentration, tests were conducted in quadruplicate in parallel to tests performed on an equal number of seeds incubated in water, taken as the negative control (blue bars).

The concentration dependence of the seed germination percentage (SG%) observed when *Lepidium sativum* L. seeds were exposed to PAA water solutions is shown in Figure 6 and Table S1. As already stated above, for each experiment carried out in PAA solutions, a parallel experiment with an equal number of seeds incubated in deionized water was performed. SG%, given by the ratio of the number of seeds germinated in the presence of the substance to be tested to the initial number of seeds (Equation (2), Section 2.3), is an indicator of acute toxicity. A lack of seed germination is, indeed, considered equivalent to mortality [12]. It is normally recognized that a substance can be classified as non-phytotoxic when the SG% ≥ 83% [16]. Since the SG% values of the tests performed with PAA solutions were invariably above 95% and always comparable to those of the negative control, it can be concluded that the PAAs considered do not elicit acute phytotoxicity, regardless of their structure, charge, and concentration, and no EC_50_ value could be determined.

**Figure 7:**
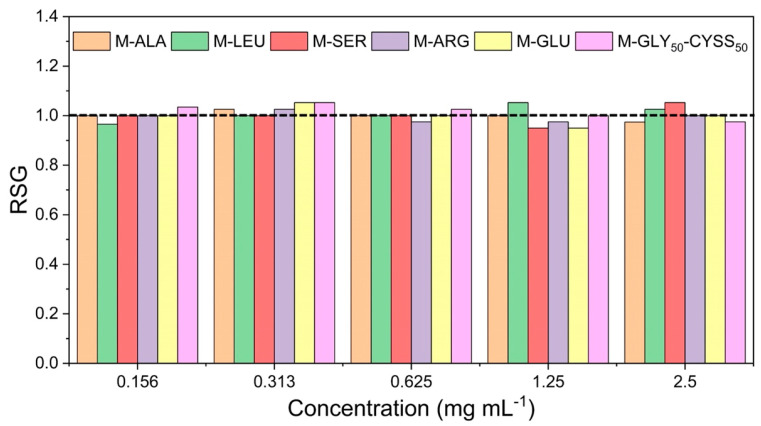
Concentration dependence of the relative seed germination (RSG) of *Lepidium sativum* L. seeds exposed to PAA solutions for 120 h. The black dashed line represents *RSG* = 1 corresponding to an equal number of seeds germinated in the test sample and in water.

Relative seed germination (RSG) values, that is the number of germinated seeds exposed to PAA solutions at different concentrations normalized to the number of germinated seeds exposed to the negative control (water), were calculated using Equation (3) (Section 2.3). The results obtained, shown in Figure 7 and Table S2, indicate that all PAAs exhibited RSG values very close to 1 all throughout the concentration range (variation range 0.95–1.05). In line with the SG% data, no acute toxicity effects were identified and there was no concentration dependence of the RSG for all PAAs.

Since the endpoint relating to the germination process is highly dependent on the energy reserves of the cotyledons, and therefore possibly less sensitive to sublethal external stress factors, endpoints relating to seedling growth are considered good indicators of chronic phytotoxicity [20]. In this study, both root and shoot length were investigated as sublethal endpoints.

**Figure 8:**
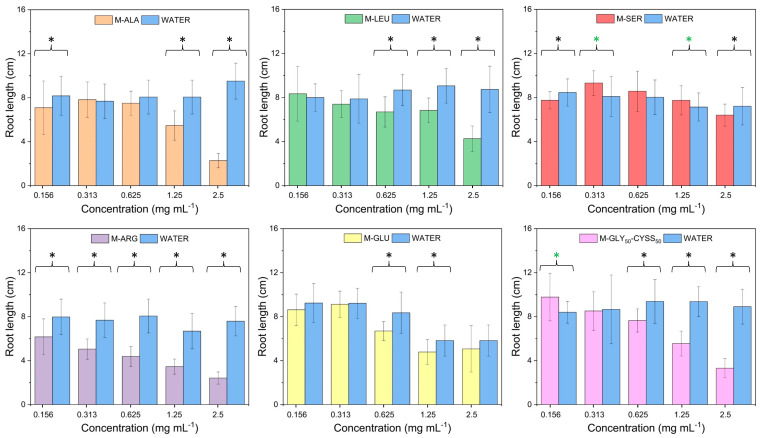
Concentration dependence of the length of *Lepidium sativum* L. roots after seed incubation in PAA aqueous solutions for 120 h (colored bars). For each PAA concentration, tests were conducted in quadruplicate in parallel to tests performed on an equal number of seeds incubated in water, which were taken as the negative control (blue bars). The results are reported as the mean ± confidence. Asterisks indicate significant differences (*p*-value < 0.05) compared to the control: the black asterisks indicate a greater length in water, the green asterisks a greater length in the PAA solution.

Root length is an indicator of the development of the root system, which is crucial for nutrient and water uptake as well as for seedling anchorage. The radical system is the most sensitive part of the plant, as it is the first organ to be exposed to contaminants in the soil or in water. Therefore, it is considered an ideal target for biomonitoring purposes [53]. The concentration dependence of *Lepidium sativum* L. root lengths, after 120 h exposure, on PAA water solutions is shown in Figure 8. For each experiment carried out in PAA solutions, a parallel experiment with an equal number of seeds incubated in deionized water was performed. For each PAA concentration, the values of the average root lengths were calculated according to the data analysis described in Section 2.5, which involves outlier definition; the evaluation of confidence as an experimental error; and the application of the *t*-test, with a *p*-value < 0.05. The latter parameter was used to evaluate significant differences compared to the results of parallel tests conducted in deionized water.

The results shown in Figure 8 indicate the different response of *Lepidium sativum* L. seeds to PAA exposure and their different concentration dependences. M-SER, featuring a moderate negative charge excess in its repeat units (−0.38 net average charge, 0.62 positive/negative charge ratio, Table 2) turned to be the most harmless of the PAAs tested. Moreover, its effect on root development did not show a clear concentration dependence. A slightly significant reduction in root elongation compared to the control was indeed observed at the maximum and minimum tested concentrations, 2.5 and 0.156 mg mL^−1^, respectively, while at intermediate concentrations, M-SER exhibited a stimulating effect, significantly so at both 0.313 and 1.5 mg mL^−1^. Unique among all the tested PAAs, M-ARG significantly and progressively reduced root elongation across the entire concentration range considered, while the control length remained almost constant. The inhibitory effect of M-ARG was attributed to the prevalence in its repeat units of positive charges (+0.29 net average charge per repeat unit, 1.29 positive/negative charge ratio, Table 2), in line with the well-known toxicity of PAAs when in their protonated form [54] and the generally recognized phytotoxicity of cationic polymers [55,56]. M-GLU, the most negatively charged PAA (−1.14 net average charge per repeat unit, 0.43 positive/negative charge ratio, Table 2), significantly reduced root length only at the two intermediate concentrations of 0.625 and 1.5 mg mL^−1^. M-ALA and M-GLY_50_-CYSS_50_, characterized by positive/negative charge ratios higher than that of M-SER, but lower than that of M-ARG (0.91 for M-ALA and 0.72 for M-GLY_50_-CYSS_50_, Table 2), significantly reduced root elongation with respect to the control at concentrations ≥ 1.25 and ≥0.625 mg mL^−1^, respectively, and drastically so at 2.5 mg mL^−1^. M-LEU, characterized by almost the same charge distribution as M-SER, but featuring a lipophilic side substituent instead of a hydrophilic one ((-CH_2_CH(CH_3_)_2_) in M-LEU and -OH in M-SER), showed a superior inhibiting effect, particularly at concentrations ≥ 0.625 mg mL^−1^, suggesting that charge distribution is not the only structural factor explaining the phytotoxicity of PAAs.

**Figure 9:**
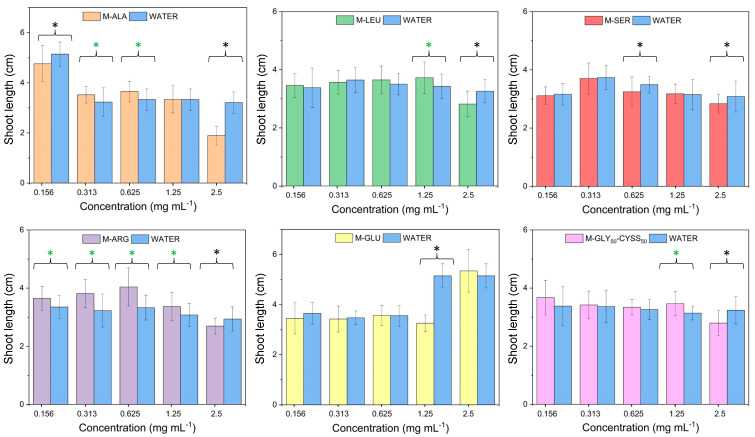
Concentration dependence of the length of *Lepidium sativum* L. shoots after seed incubation in PAA aqueous solutions for 120 h (colored bars). For each PAA concentration, tests were conducted in quadruplicate in parallel to tests performed on an equal number of seeds incubated in water, which were taken as the negative control (blue bars). The results are reported as the mean ± confidence. Asterisks indicate significant differences (*p*-value < 0.05) compared to the control: black asterisks indicate a greater length in water, and green asterisks a greater length in the PAA solution.

The concentration dependence of *Lepidium sativum* L. shoot lengths, the second bioindicator considered, after 120 h exposure to PAA water solutions is shown in Figure 9. Overall, the shoot growth was not significantly affected by PAA exposures. A significant reduction in the shoot length was indeed observed only at the highest tested concentration, 2.5 mg mL^−1^, except for M-GLU, which induced a significant shoot length reduction only at 1.25 mg mL^−1^. Interestingly, the shoot length of seedlings grown in M-ARG solutions, which exhibited the greatest inhibitory effect of all, based on the root length indicator, indicated an M-ARG stimulating effect from 0.156 to 1.25 mg mL^−1^. These observations align with literature reports that underline how toxic substances can influence radicle and shoot development differently [28].

**Figure 10:**
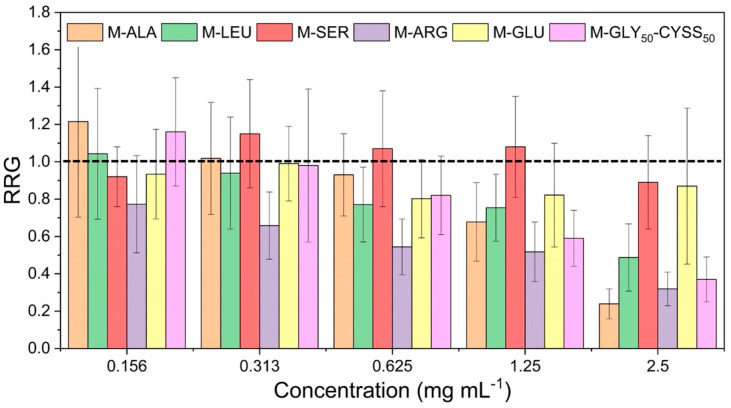
Concentration dependence of the relative radicle growth of *Lepidium sativum* L. seedlings in PAA water solutions after 120 h. The results are reported as the mean ± confidence. The black dashed line represents *RRG* = 1 corresponding to an equal radicle length in the test sample and in water.

To better analyze the effect of PAAs on the radicle growth of *Lepidium sativum* L., the Relative Root Growth (RRG) was calculated as the ratio of the length of roots grown in PAA solutions to that of roots grown in water (Equation (4), Section 2.3). A chemical compound is normally classified as a plant fertilizer when its RRG ≥ 1, while it is classified as an inhibitor when its RRG < 1. Figure 10 and Table S3 show the RRG values after 120 h of seed exposure to PAA water solutions at different concentrations. Notably, all PAAs had an inhibiting effect at 2.5 mg mL^−1^, but the RRG was above 0.9 in the case of M-SER and M-GLU. M-SER’s stimulating effect was observed in the concentration range 0.156–1.25 mg mL^−1^ and M-GLY_50_-CYSS_50_ and M-ALA’s at 0.156 mg mL^−1^. A regular increase in the inhibitory effect with the concentration of M-ALA, M-LEU, M-ARG, and M-GLY_50_-CYSS_50_ was observed. Not unexpectedly, M-ARG showed an inhibitory effect at all tested concentrations, consistent with the results of the root length measurements.

The seed germination test can be used to classify the overall performance of a chemical compound through the germination index, GI (Equation (5), Section 2.3). This index combines measurements of both seed germination rate and root elongation, providing a comprehensive and integrative assessment of plant development. A GI < 0.5 indicates high phytotoxicity, values in the range 0.5 < GI < 0.8 suggest moderate phytotoxicity, and values in the range 0.8 < GI < 1 indicate a lack of phytotoxicity and only a moderate inhibitory effect. If the GI > 1 the test compound can be classified as a phytonutrient or phytostimulant [57].

**Figure 11:**
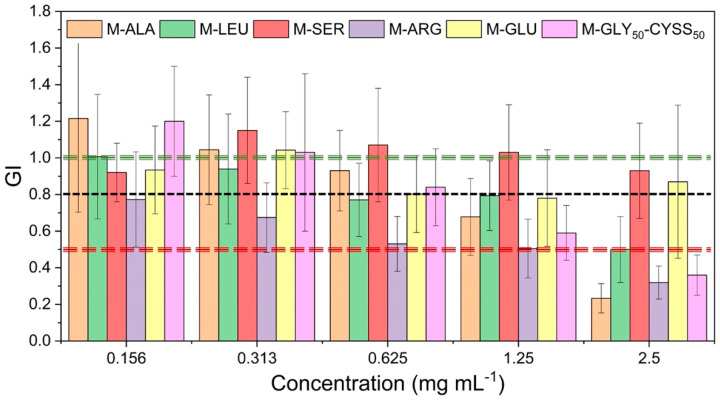
Concentration dependence of the germination index of *Lepidium sativum* L. seeds exposed to PAA water solutions for 120 h. The red (*GI* = 0.5), black (*GI* = 0.8) and green (*GI* = 1.0) dashed lines represent threshold values of different phytotoxic levels. In detail: *GI* < 0.5 corresponds to high phytotoxicity; 0.5 < *GI* < 0.8 corresponds to moderate phytotoxicity; 0.8 < *GI* < 1 corresponds to moderate inhibitory effect; *GI* > 1 corresponds to phytostimulation.

The concentration dependence of the GI after seed incubation in PAA solutions for 120 h is shown in Figure 11 and Table S4. The collected results indicate that most PAAs are phytotoxic at the highest tested concentration, 2.5 mg mL^−1^, except M-SER and M-GLU, which show only a modest inhibitory effect. In line with the RRG data, M-SER exhibited a clear stimulating effect from 0.313 to 1.25 mg mL^−1^. The GI values confirmed that the most toxic PAA of all is M-ARG, the prevailingly cationic one, which features a GI < 0.8 across the whole concentration range tested and a GI equal or less than 0.5 above 0.625 mg mL^−1^. M-GLU, the most anionic of all PAAs, is non phytotoxic at all concentrations and slightly stimulating at 0.313 mg mL^−1^. M-LEU has borderline behavior at 0.625 and 1.25 mg mL^−1^, while M-ALA and M-GLY_50_-CYSS_50_, featuring a medium–high positive/negative charge value per repeat unit (0.66 and 0.72, respectively), are both phytotoxic at concentrations above 1.25 mg mL^−1^ and show similar general behavior across the whole concentration rage.

The usefulness of the GI values lies in the fact that they allowed the studied PAAs to be classified based on their inhibitory effect on seed germination. Furthermore, they allowed us to correlate the observed inhibitory effect with the PAA charge distribution, demonstrating the validity of the test not only in assessing the phytotoxicity of water-soluble polymers but also in correlating phytotoxicity to the structural features of the test compounds. However, when assessing the phytotoxicity of the PAAs under investigation, it should be kept in mind that the concentration range investigated in this work significantly exceeded the maximum concentration recommended by the EPA guidelines, namely 1 mg mL^−1^ [12].

Moreover, the mildness of the effect of the investigated PAAs on the environment, as deducible from the seed germination test, stands alone among those generally reported for polymeric materials. For instance, the detrimental effect of microplastics on plant growth, such as their interference with the absorption and transportation of nutrients, their reduction or delay in seed germination by inhibiting imbibition of water, and their alteration of root and shoot growth, have been well documented [58].

As a final remark, it may be observed that, thanks to their water solubility, prevailing negative charge, and high charge density, anionic PAAs with a GI > 0.8 can be considered promising organic soil conditioners similar to other organic polymers already used in agriculture [59].

## 4. Conclusions

The objective of this study was to evaluate the versatility of the seed germination test, an inexpensive and quick test that can be used for screening large numbers of samples, in assessing the phytotoxicity of water-soluble polymers. To this end, a set of α-amino acid-derived polyamidoamines currently being investigated as flame retardants for cotton was subjected to seed germination tests using *Lepidium sativum* L. as the model system. The seeds were incubated in polyamidoamine water solutions with concentrations ranging from 0.156 to 2.5 mg mL^−1^ for 120 h at 25 ± 1 °C. Notably, the maximum concentration tested was higher than the maximum concentration indicated in the EPA guidelines for seed germination tests [15]; this was chosen to stress the test and possibly determine the EC_50_ values of the tested polymers. Different parameters were chosen as phytotoxicity endpoints, including the seed germination percentage, relative seed germination, root and shoot length, relative radical growth, and germination index. Additionally, the germination kinetics of *Lepidium sativum* L. seeds was monitored by optical microscopy to identify possible inhibitory effects on the early stages of germination. None of the polymers exhibited acute toxicity since their seed germination percentage was invariably > 95% and their germination times were substantially unchanged. Radicle and shoot length proved to be useful biomarkers for monitoring the inhibitory effects of polymers, although radicle length was revealed to be more sensitive. The phytocompatibility of the PAAs was classified based on their GI values. The predominantly anionic serine and glutamic acid derivatives proved to be phytocompatible at all concentrations, whereas the predominantly cationic arginine derivative exhibited an increasing inhibitory effect from 0.313 mg mL^−1^. The time to rupture of the testa tegument in the seeds exposed to M-ARG was anomalous. Overall, the reported results proved the validity of the test for assessing the phytotoxicity of water-soluble polymers and correlating this to their structural features.

## Figures and Tables

**Table 1:** Experimental conditions adopted in the synthesis of PAAs.

PAA ^(a)^	α-Amino Acid	α-Amino Acid (g; mmol)	LiOH·H_2_O (g; mmol)	Time (Days)
M-ALA [39]	alanine	2.90; 32.23	1.38; 32.23	5
M-LEU	leucine	4.25; 32.07	1.37; 32.00	10
M-SER	serine	3.41; 32.12	1.38; 32.23	5
M-ARG [40]	arginine	5.65; 32.11	-	9
M-GLU [41]	glutamic acid	4.83; 32.17	2.74; 64.00	9
M-GLY_50_-CYSS_50_ [42] ^(b)^	glycine	1.23; 16.06	4.12; 96.22	2
	cystine	3.89; 16.03		

^(a)^ PAAs were synthesized from *N*,*N*′-methylenebisacrylamide (5 g, 32.11 mmol, coded as M) and the α-amino acids listed in the second column, in 10 mL H_2_O at 25 ± 1 °C. ^(b)^ Ultrafiltration was performed at pH 7.5.

**Table 2:** Average charge of PAAs’ repeat units.

PAA ^(a)^	*pK_a_* Values	IP ^(b)^	Net Charge at pH 7 ^(c)^	Positive/Negative Charge ^(d)^
M-ALA [39]	*pK_a-COOH_* = 2.12 *pK_a-NR_*_3_ = 8.13	5.1	−0.09	0.91
M-LEU [39]	*pK_a-COOH_* = 2.11 *pK_a-NR_*_3_ = 7.37	4.8	−0.34	0.66
M-SER	*pK_a-COOH_* = 2.14 *pK_a-NR_*_3_ = 7.08	4.6	−0.38	0.62
M-ARG [40]	*pK_a-COOH_* = 2.2 *pK_a-NR_*_3_ = 6.4 *pK_a-guanidine_* > 10	9.7	+0.29	1.29
M-GLU [41]	*pK_a-COOH,_*_1_ = 2.32 *pK_a-COOH,_*_2_ = 4.28 *pK_a-NR_*_3_ = 7.78	3.3	−1.14	0.43
M-GLY_50_-CYSS_50_	-	4.9	−0.35	0.72
M-GLY [36]	*pK_a-COOH_* = 1.9 *pK_a-NR_*_3_ = 7.7	4.8	−0.39	0.61
M-CYSS [47]	*pK_a-NR_*_3,1_ = 2.4 *pK_a-NR_*_3,2_ = 4.0 *pK_a-COOH,_*_1_ = 8.2 *pK_a-COOH,_*_2_ = 12.7	5.0	−0.31	0.84

^(a)^ PAA codes are specified in Table 1. ^(b)^ Isoelectric point. ^(c)^ Data calculated from the charge distribution shown in Figure 1. ^(d)^ Data calculated at pH 7.5.

## Data Availability

The raw/processed data required to reproduce the above findings cannot be shared at this time as the data also form part of an ongoing study.

## Supplementary Materials

The following supporting information can be downloaded at: https://www.mdpi.com/article/10.3390/polym16121744/s1, Figure S1: Data distribution set of plant length measurements when *Lepidium sativum* L. seeds were exposed to water, as an example of outliers; Figures S2–S7: ^1^H-NMR spectra of M-ALA, M-LEU, M-SER, M-ARG, M-GLU, and M-GLY_50_-CYSS_50_; Figures S8–S13: Speciation diagrams of M-ALA, M-LEU, M-SER, M-ARG, M-GLU, and M-GLY_50_-CYSS_50_; Figure S14: Image of a set of 10 *Lepidium sativum* L. seeds exposed to deionized water as a negative control; Figures S15–S18: *Lepidium sativum* L. seedling growth after seed exposure to 0.156, 0.313, 0.625, and 1.25 mg mL^−1^ PAA solutions, after an incubation time of 120 h. Table S1: Germination percentage of *Lepidium sativum* seeds exposed to PAA water solutions at different concentrations after an incubation time of 120 h; Table S2: Relative seed germination of *Lepidium sativum* L. seeds exposed to PAA water solutions at different concentrations after an incubation time of 120 h; Table S3: Relative radicle growth of *Lepidium sativum* L. seedlings in PAA water solutions at different concentrations after an incubation time of 120 h; Table S4: Germination index of *Lepidium sativum* L. seeds exposed to PAA water solutions at different concentrations after an incubation time of 120 h.

## Abbreviation

**Abbreviation**	**Meaning**
EC_50_	50% effective concentration
EPA	Environmental protection agency
GI	Germination index
M	*N*,*N*′-Methylenebisacrylamide
M-ALA	M-alanine-derived polyamidoamine
M-ARG	M-arginine-derived polyamidoamine
M-CYSS	M-cystine-derived polyamidoamine
M-GLU	M-glutamic acid-derived polyamidoamine
M-GLY	M-glycine-derived polyamidoamine
M-GLY_50_-CYSS_50_	M-glycine-cystine-derived polyamidoamine
M-LEU	M-leucine-derived polyamidoamine
M-SER	M-serine-derived polyamidoamine
PAA	Polyamidoamine
REACH	Regulation, evaluation, authorization, and restriction of chemicals
RRG	Relative radicle growth
RSG	Relative seed germination
SG%	Seed germination percentage
