# Supplementary material for: The Seed Germination Test as a Valuable Tool for the Short-Term Phytotoxicity Screening of Water-Soluble Polyamidoamines
Source: Polymers (Basel). 2024 Jun 19;16(12):1744. doi: 10.3390/polym16121744 (PMC11207469; doi:10.3390/polym16121744)
Supplement: Supplementary file 1 [file polymers-16-01744-s001.zip › polymers-3039928-supplementary.pdf]

SUPPLEMENTARY MATERIALS

Seed germination test as a valuable tool for the short-term toxicity screening of water-soluble polyamidoamines

Elisabetta Ranucci, Sofia Treccani, Paolo Ferruti, Jenny Alongi*
Dipartimento di Chimica, Università degli Studi di Milano, Via C. Golgi 19, 20133
*corresponding author: jenny.alongi@unimi.it; tel.: +390250314108

Pages S1-S15

Figure S1. Data distribution set of plant length measurements when *Lepidium sativum* seeds were exposed to water, as an example of outliers.

Figures S2-S7. ^{1}H-NMR spectra of M-ALA, M-LEU, M-SER, M-ARG, M-GLU and M-GLY_{50}-$CYSS_{50}$.

Figures S8-S13. Speciation diagrams of M-ALA, M-LEU, M-SER, M-ARG, M-GLU and M-GLY_{50}-$CYSS_{50}$.

Figure S14. Image of a set of 10 *Lepidium sativum* seeds exposed to deionized water as a negative control.

Figures S15-S18. *Lepidium sativum* seedling growth after seed exposure to 0.156, 0.313, 0.625 and 1.25 mg mL^{-1} PAA solutions after an incubation time of 120 h.

Table S1. Percent germination of *Lepidium sativum* seeds exposed to PAA water solutions at different concentrations after an incubation time of 120 h.

Table S2. Relative seed germination of *Lepidium sativum* seeds exposed to PAA water solutions at different concentrations after an incubation time of 120 h.

Table S3. Relative Radicle Growth of *Lepidium sativum* seedlings in PAA water solutions at different concentrations after an incubation time of 120 h.

Table S4. Germination index of *Lepidium sativum* seeds exposed to PAA water solutions at different concentrations after an incubation time of 120 h.

Evaluation of outlier data

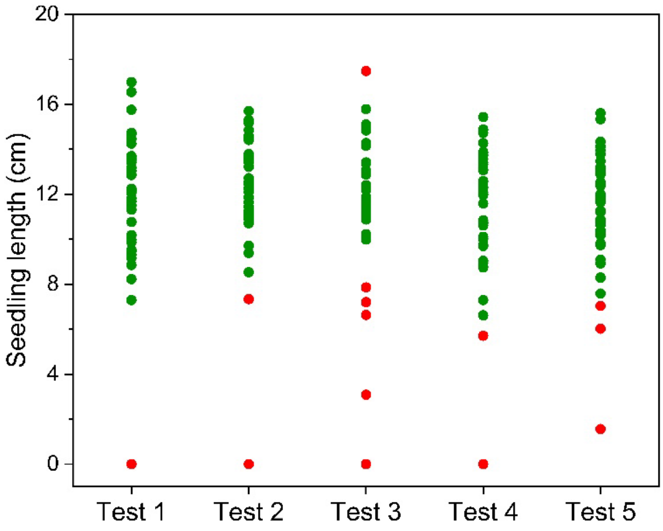

Figure S1. Data distribution set of plant length measurements when *Lepidium sativum* seeds were exposed to water, as an example of outliers. The red circles represent outliers, the green ones represent data considered for the calculation of the average values.

^{1}H-NMR characterization of PAAs

The chemical structure of M-GLY, M-GLU and M-GLY$_{50}$-CYSS$_{50}$ was assessed by ^{1}H-NMR, collecting spectra in D_2O at pH 4.0 and at 25 °C using a Bruker Advance DPX-400 NMR spectrometer (Milan, Italy) operating at 400.13 MHz. Parameters: scan number 32, relaxation delay, *d1*, 10.0 s, receiver gain automatically measured and set by the instrument.

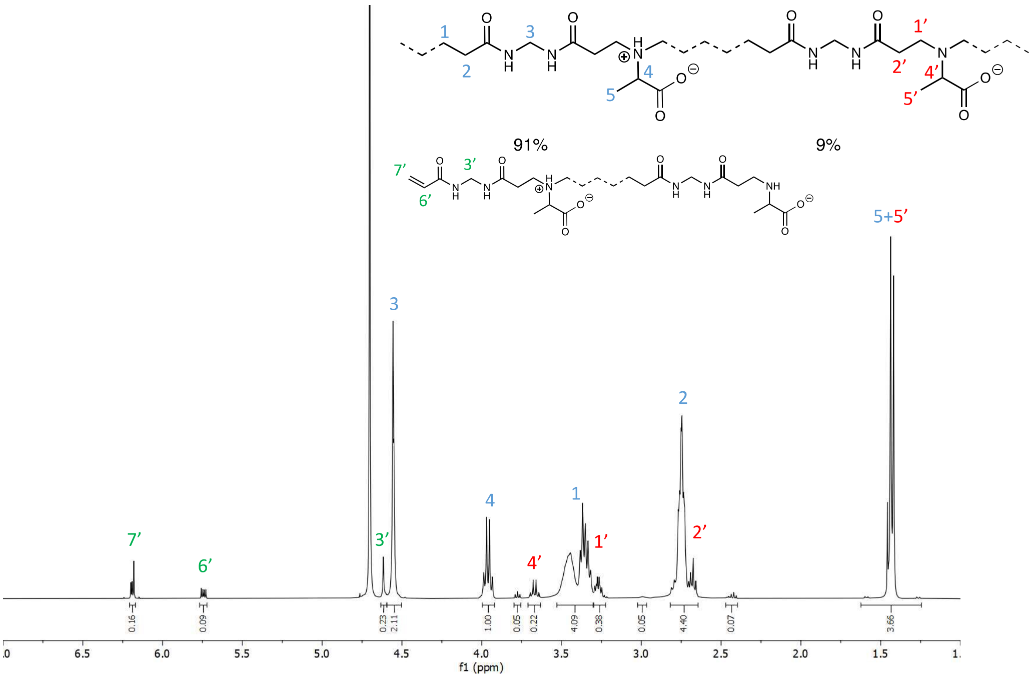

Figure S2. ^{1}H-NMR spectrum of M-ALA.

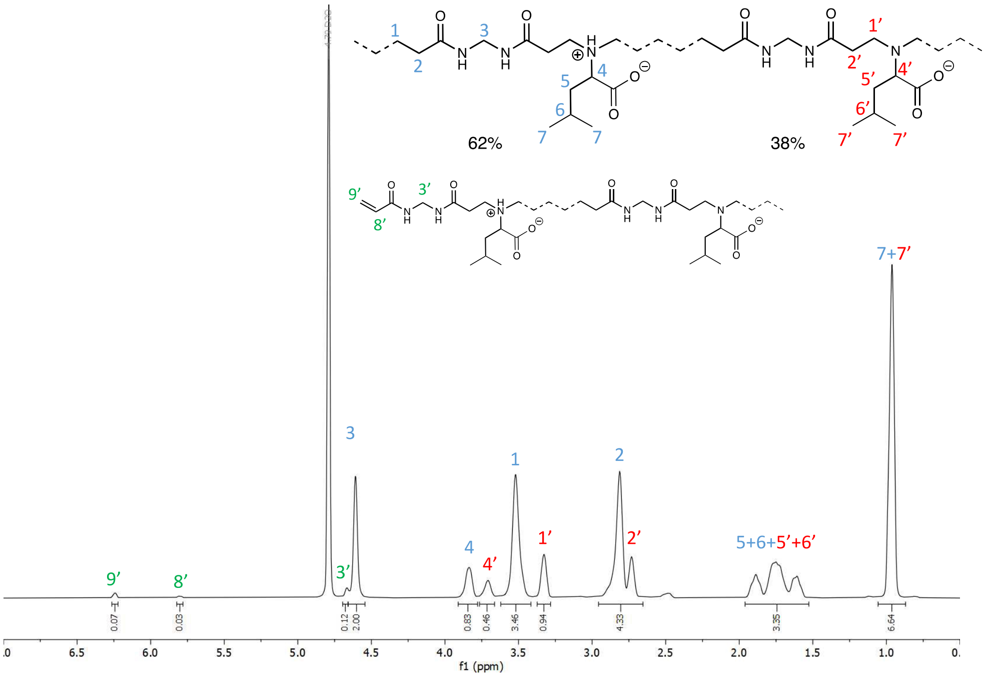

Figure S3. ^{1}H-NMR spectrum of M-LEU.

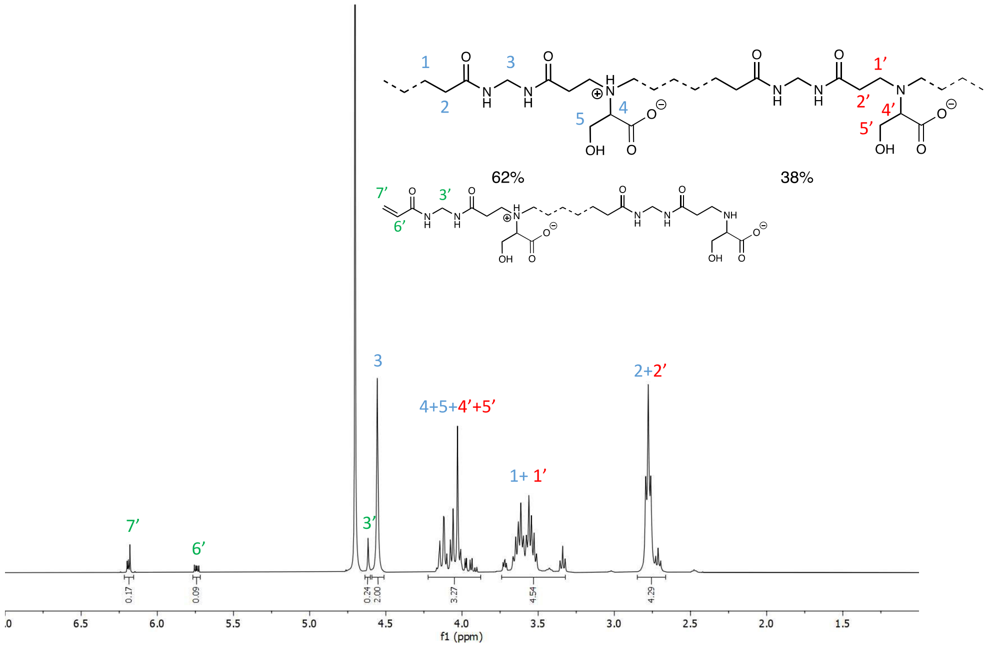

Figure S4. ^{1}H-NMR spectrum of M-SER.

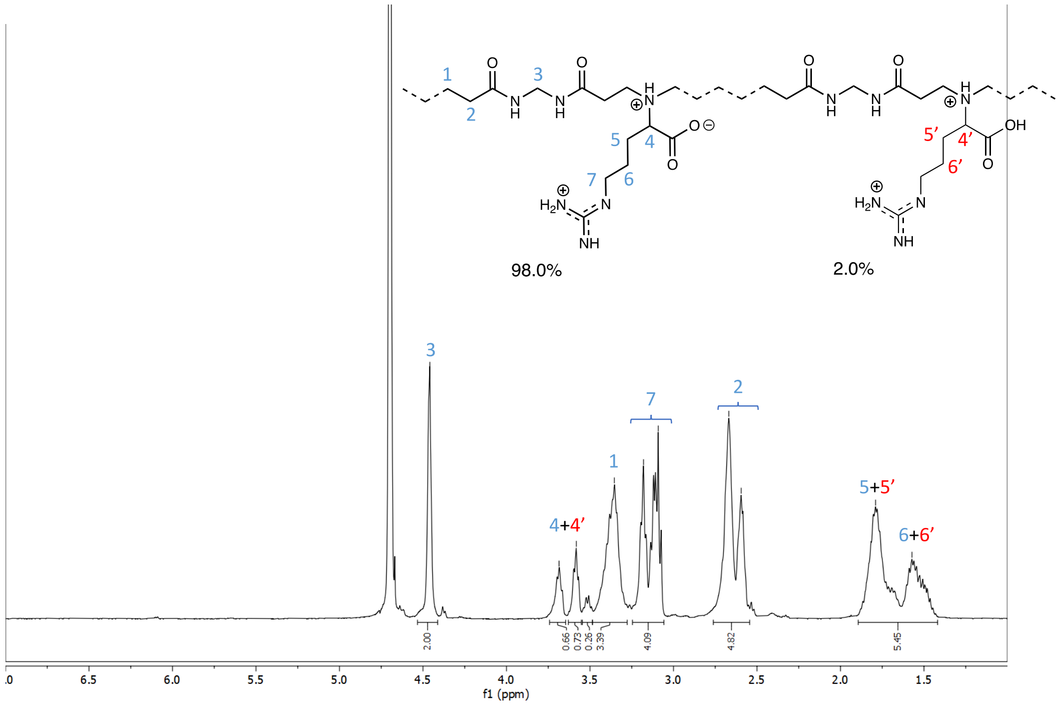

Figure S5. ^{1}H-NMR spectrum of M-ARG.

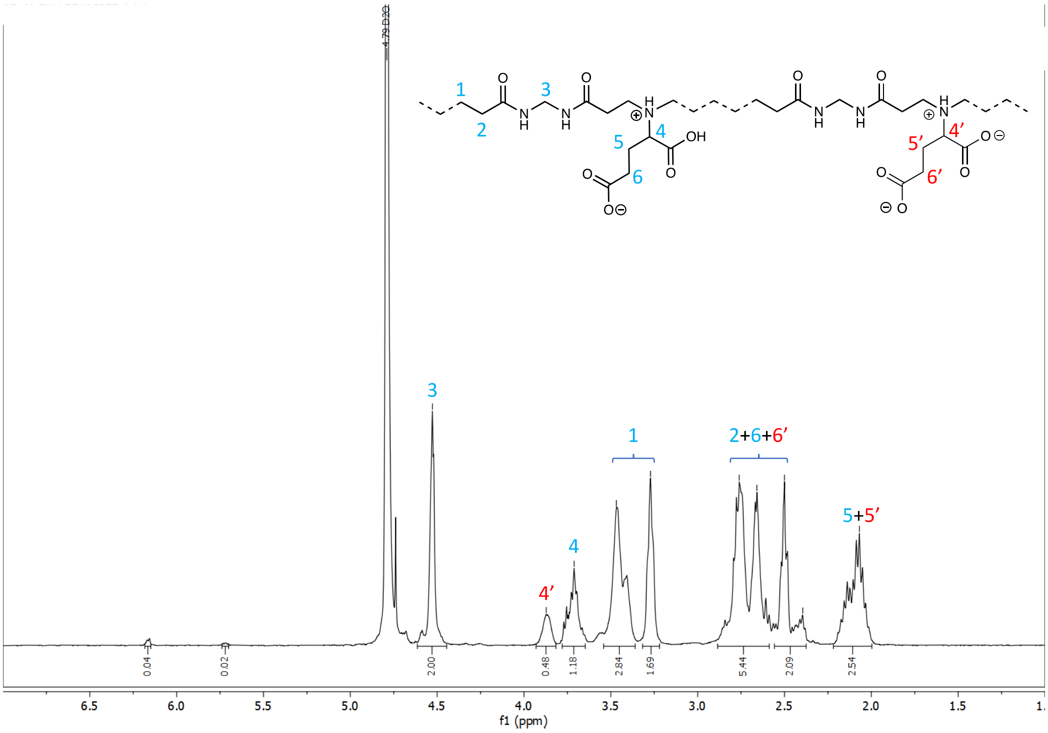

Figure S6. ^{1}H-NMR spectrum of M-GLU.

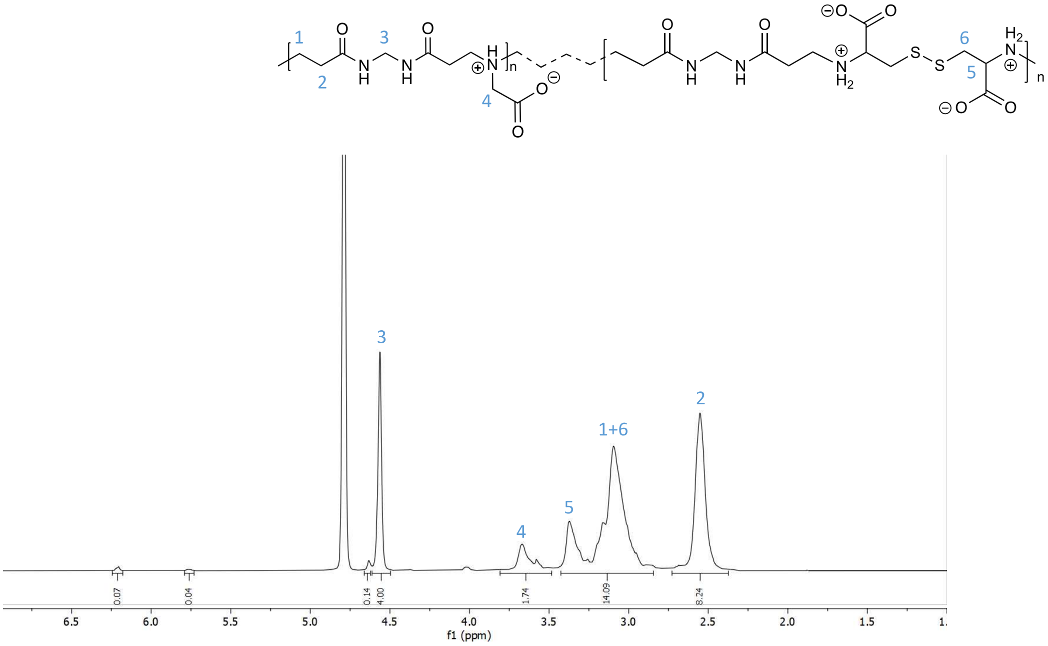

Figure S7. ^{1}H-NMR spectrum of M-GLY$_{50}$-CYSS$_{50}$.

Determination of the *pKa* values of PAAs and speciation diagrams

M-ALA: the pK_a values were calculated in a previous work [1]. Speciation diagrams (Figure S8) were obtained by plotting the concentration fractions (α) of the different ionic species as a function of pH. The concentration fractions of M-ALA were calculated according to the following equations:

- Mass balance: $C_0 = C_{L+} + C_{L0} + C_{L-}$
- Equilibrium constants: $K_{a1} = \frac{C_{L0}\, C_{H+}}{C_{L+}}$; $K_{a2} = \frac{C_{L-}\, C_{H+}}{C_{L0}}$
- Concentration fractions:

$$C_{L+} = \frac{C_0\, {C_{H+}}^2}{{C_{H+}}^2 + K_{a1}\, C_{H+} + K_{a1}K_{a2}} = \frac{C_0\, {C_{H+}}^2}{D} \longrightarrow \boldsymbol{\alpha}_{L+} = \frac{C_{L+}}{C_0} = \frac{{C_{H+}}^2}{D}$$

$$C_{L0} = \frac{C_0\, K_{a1}\, C_{H+}}{{C_{H+}}^2 + K_{a1}\, C_{H+} + K_{a1}K_{a2}} = \frac{C_0\, K_{a1}\, C_{H+}}{D} \longrightarrow \boldsymbol{\alpha}_{L0} = \frac{C_{L0}}{C_0} = \frac{K_{a1}\, C_{H+}}{D}$$

$$C_{L-} = \frac{C_0\, K_{a1}K_{a2}}{{C_{H+}}^2 + K_{a1}\, C_{H+} + K_{a1}K_{a2}} = \frac{C_0\, K_{a1}K_{a2}}{D} \longrightarrow \boldsymbol{\alpha}_{L-} = \frac{C_{L-}}{C_0} = \frac{K_{a1}K_{a2}}{D}$$

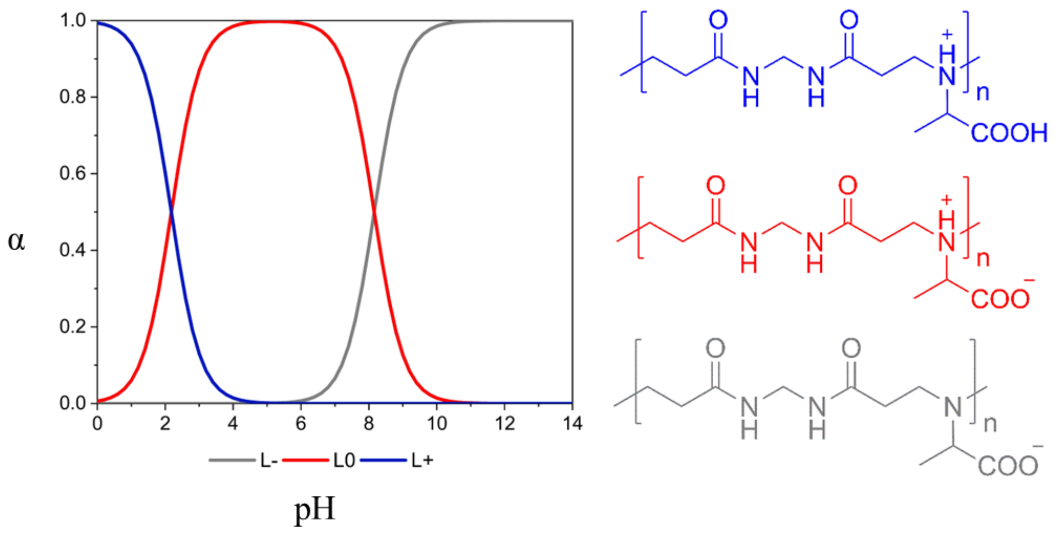

Figure S8. Speciation diagram and chemical structures of the ionized repeat units of M-ALA.

M-LEU: the pK_a values were calculated in a previous work [1]. Speciation diagrams (Figure S9) were obtained by plotting the concentration fractions (α) of the different ionic species as a function of pH. The concentration fractions of M-LEU were calculated using the same equations used for M-ALA.

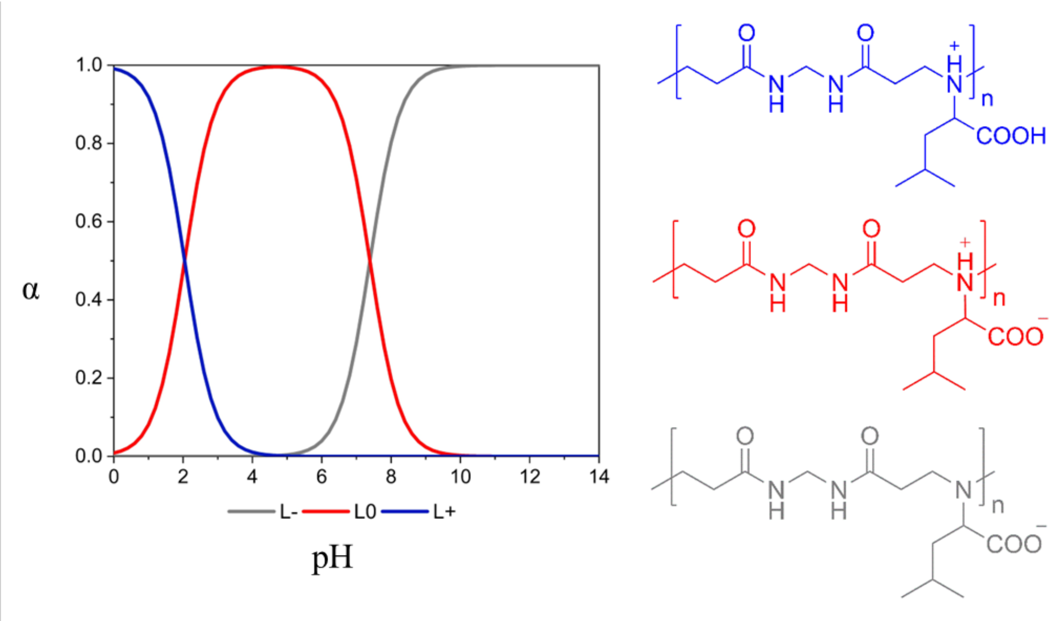

Figure S9. Speciation diagram and chemical structures of the ionized repeat units of M-LEU.

M-SER: the pK_a values were calculated as described for M-ALA [1]. Speciation diagrams (Figure S10) were obtained by plotting the concentration fractions (α) of the different ionic species as a function of pH. The concentration fractions of M-SER were calculated using the same equations used for M-ALA.

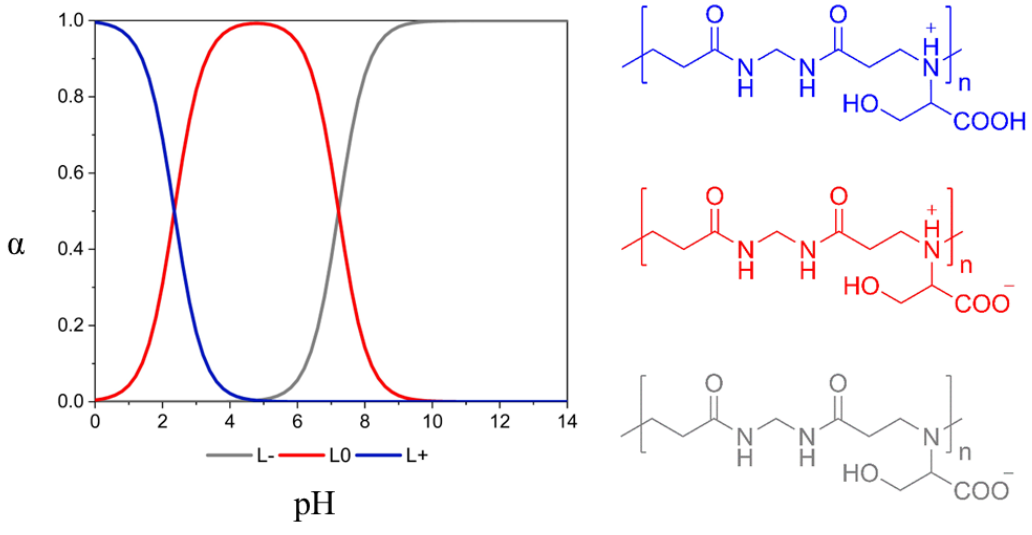

Figure S10. Speciation diagram and chemical structures of the ionized repeat units of M-SER.

M-ARG: the pK_a values were calculated in a previous work [2]. Speciation diagrams (Figure S11) were obtained by plotting the concentration fractions (α) of the different ionic species as a function of pH. The concentration fractions of M-ARG were calculated according to the following equations:

- Mass balance: $C_0 = C_{L2+} + C_{L+} + C_{L0} + C_{L-}$
- Equilibrium constants: $K_{a1} = \frac{C_{L+}\,C_{H+}}{C_{L2+}}$; $K_{a2} = \frac{C_{L0}\,C_{H+}}{C_{L+}}$; $K_{a3} = \frac{C_{L-}\,C_{H+}}{C_{L0}}$;
- Concentration fractions:

$$C_{L2+} = \frac{C_0\,{C_{H+}}^3}{{C_{H+}}^3 + {C_{H+}}^2 K_{a1} + C_{H+} K_{a1} K_{a2} + K_{a1} K_{a2} K_{a3}} = \frac{C_0\,{C_{H+}}^3}{D} \rightarrow \boldsymbol{\alpha}_{L2+} = \frac{C_{L2+}}{C_0} = \frac{{C_{H+}}^3}{D}$$

$$C_{L+} = \frac{C_0 {C_{H+}}^2 K_{a1}}{D} \rightarrow \boldsymbol{\alpha}_{L+} = \frac{C_{L+}}{C_0} = \frac{{C_{H+}}^2 K_{a1}}{D}$$

$$C_{L0} = \frac{C_0\,C_{H+}\,K_{a1} K_{a2}}{D} \rightarrow \boldsymbol{\alpha}_{L0} = \frac{C_{L0}}{C_0} = \frac{C_{H+}\,K_{a1} K_{a2}}{D}$$

$$C_{L-} = \frac{C_0\,K_{a1} K_{a2} K_{a3}}{D} \rightarrow \boldsymbol{\alpha}_{L-} = \frac{C_{L-}}{C_0} = \frac{K_{a1} K_{a2} K_{a3}}{D}$$

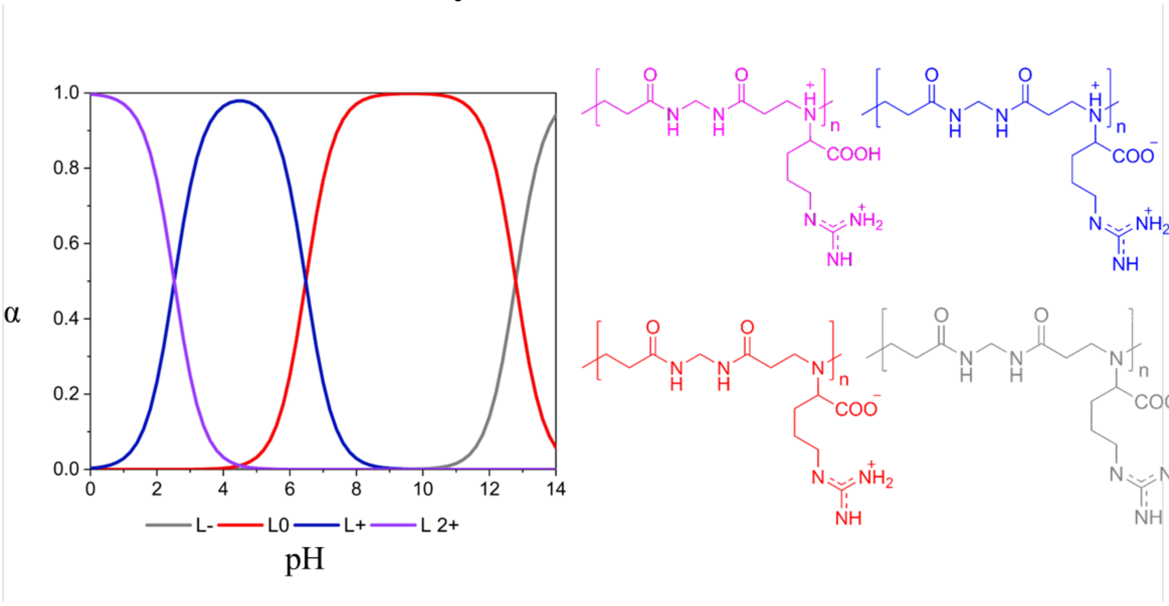

Figure S11. Speciation diagram and chemical structures of the ionized repeat units of M-ARG.

M-GLU: the pK_a values were calculated in a previous work [3]. Speciation diagrams (Figure S12) were obtained by plotting the concentration fractions (α) of the different ionic species as a function of pH. The concentration fractions of M-GLU were calculated according to the following equations:

- Mass balance: $C_0 = C_{L+} + C_{L0} + C_{L-} + C_{L2-}$
- Equilibrium constants: $K_{a1} = \frac{C_{L0}\, C_{H+}}{C_{L+}}$; $K_{a2} = \frac{C_{L-}\, C_{H+}}{C_{L0}}$; $K_{a3} = \frac{C_{L2-}\, C_{H+}}{C_{L-}}$
- Concentration fractions:

$$C_{L+} = \frac{C_0\, C_{H+}^{\;3}}{C_{H+}^{\;3} + C_{H+}^{\;2} K_{a1} + C_{H+}\, K_{a1}K_{a2} + K_{a1}K_{a2}K_{a3}} = \frac{C_0\, C_{H+}^{\;3}}{D} \longrightarrow \boldsymbol{\alpha}_{L+} = \frac{C_{L+}}{C_0} = \frac{C_{H+}^{\;3}}{D}$$

$$C_{L0} = \frac{C_0\, C_{H+}^{\;2} K_{a1}}{D} \longrightarrow \boldsymbol{\alpha}_{L0} = \frac{C_{L0}}{C_0} = \frac{C_{H+}^{\;2} K_{a1}}{D}$$

$$C_{L-} = \frac{C_0\, C_{H+}\, K_{a1}K_{a2}}{D} \longrightarrow \boldsymbol{\alpha}_{L-} = \frac{C_{L-}}{C_0} = \frac{C_{H+}\, K_{a1}K_{a2}}{D}$$

$$C_{L2-} = \frac{C_0\, K_{a1}K_{a2}K_{a3}}{D} \longrightarrow \boldsymbol{\alpha}_{L2-} = \frac{c_{L2-}}{c_0} = \frac{K_{a1}K_{a2}K_{a3}}{D}$$

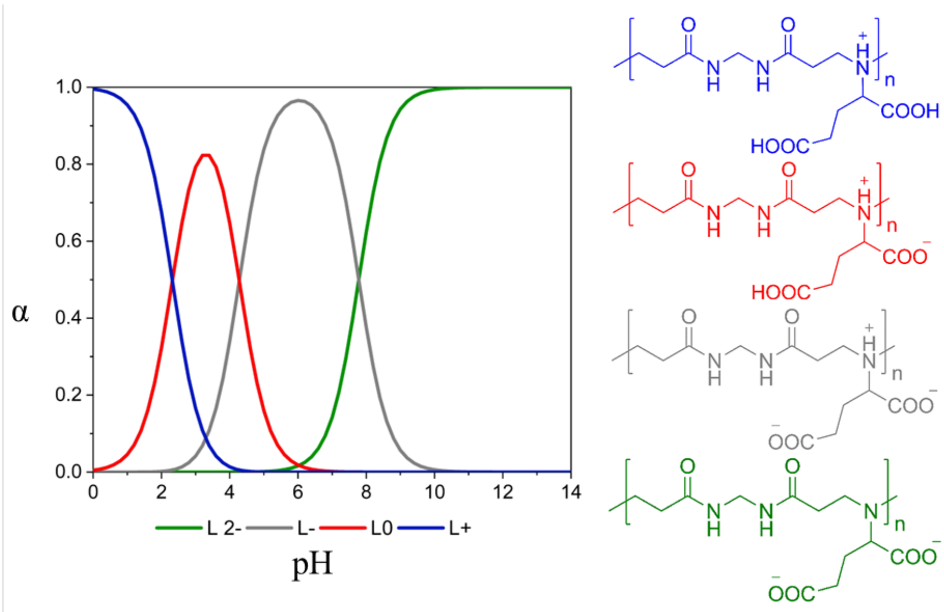

Figure S12. Speciation diagram and chemical structures of the ionized repeat units of M-GLU.

M-CYSS: the pK_a values were calculated in a previous work [3]. Speciation diagrams (Figure S13) were obtained by plotting the concentration fractions (α) of the different ionic species as a function of pH. The concentration fractions of M-CYSS were calculated according to the following equations:

- Mass balance: $C_0 = C_{L2+} + C_{L+} + C_{L0} + C_{L-} + C_{L2-}$
- Equilibrium constants: $K_{a1} = \frac{C_{L+}\,C_{H+}}{C_{L2+}}$; $K_{a2} = \frac{C_{L0}\,C_{H+}}{C_{L+}}$; $K_{a3} = \frac{C_{L-}\,C_{H+}}{C_{L0}}$; $K_{a4} = \frac{C_{L2-}\,C_{H+}}{C_{L-}}$
- Concentration fractions:

$$C_{L2+} = \frac{C_0\,C_{H+}^{\;4}}{C_{H+}^{\;4} + C_{H+}^{\;3}K_{a1} + C_{H+}^{\;2}\,K_{a1}K_{a2} + C_{H+}K_{a1}K_{a2}K_{a3} + K_{a1}K_{a2}K_{a3}K_{a4}}$$

$$= \frac{C_0\,C_{H+}^{\;4}}{D} \rightarrow \boldsymbol{\alpha}_{L2+} = \frac{c_{L2+}}{c_0} = \frac{C_{H+}^{\;4}}{D}$$

$$C_{L+} = \frac{C_0 C_{H+}^{\;3}K_{a1}}{D} \rightarrow \boldsymbol{\alpha}_{L+} = \frac{c_{L+}}{c_0} = \frac{C_{H+}^{\;3}K_{a1}}{D}$$

$$C_{L0} = \frac{C_0\,C_{H+}^{\;2}\,K_{a1}K_{a2}}{D} \rightarrow \boldsymbol{\alpha}_{L0} = \frac{c_{L0}}{c_0} = \frac{C_{H+}^{\;2}\,K_{a1}K_{a2}}{D}$$

$$C_{L-} = \frac{C_0\,C_{H+}K_{a1}K_{a2}K_{a3}}{D} \rightarrow \boldsymbol{\alpha}_{L-} = \frac{c_{L-}}{c_0} = \frac{C_{H+}K_{a1}K_{a2}K_{a3}}{D}$$

$$C_{L2-} = \frac{C_0\,K_{a1}K_{a2}K_{a3}K_{a4}}{D} \rightarrow \boldsymbol{\alpha}_{L2-} = \frac{c_{L2-}}{c_0} = \frac{K_{a1}K_{a2}K_{a3}K_{a4}}{D}$$

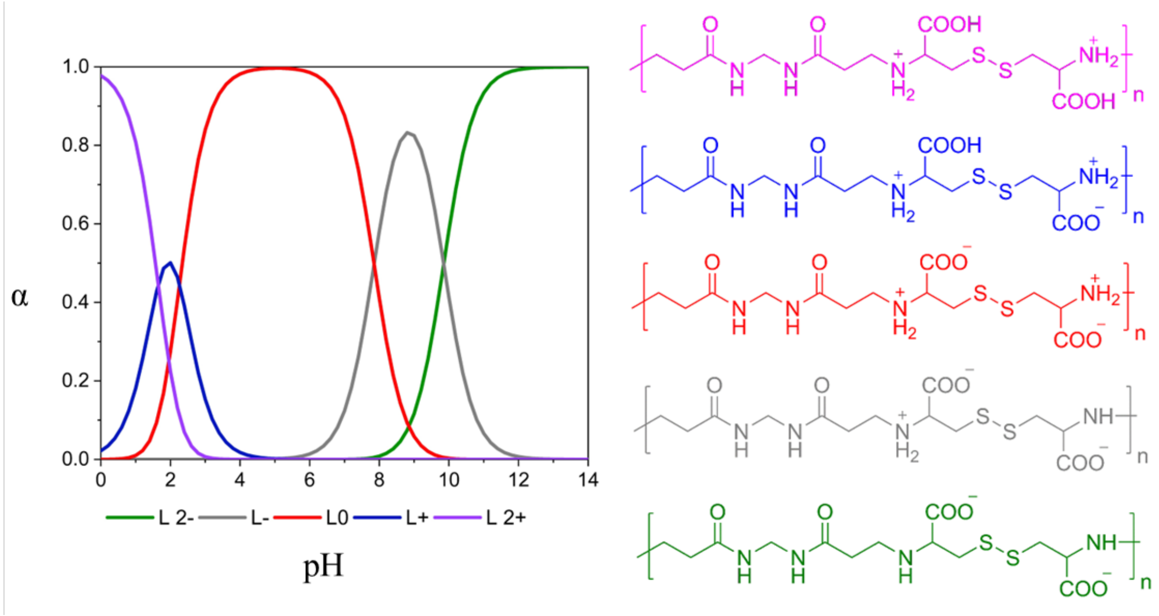

Figure S13. Speciation diagram and chemical structures of the ionized repeat units of M-CYSS.

Seed germination test

The results of the seed germination test were reported as: seed germination percentage (SG %), relative seed germination (RSG), relative radicle growth (RRG) and germination index (GI), obtained using Equations (1) - (4) [4]:

$$SG\% = \frac{Number\ of\ germinated\ seeds}{Number\ of\ all\ tested\ seeds} x100 \qquad \text{Eq. 1}$$

$$RSG = \frac{Number\ of\ germinated\ seeds\ exposed\ to\ sample\ to\ test}{Number\ of\ germinated\ seeds\ exposed\ to\ negative\ control} \qquad \text{Eq. 2}$$

$$RRG = \frac{Length\ of\ germinated\ seeds\ exposed\ to\ sample\ to\ test}{Length\ of\ germinated\ seeds\ exposed\ to\ negative\ control} \qquad \text{Eq. 3}$$

$$GI = RSG\ x\ RRG \qquad \text{Eq. 4}$$

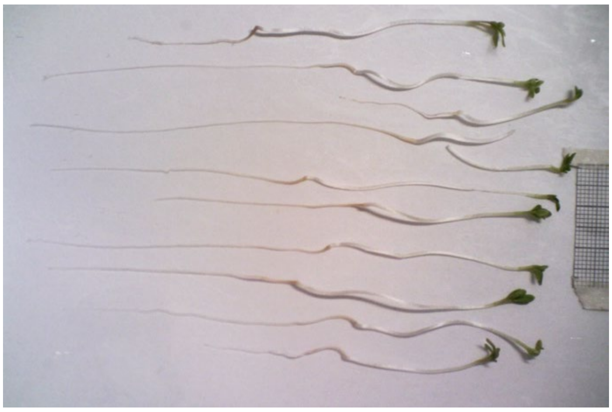

Figure S14. Image of a set of 10 *Lepidium sativum* seedlings obtained by exposing seeds to deionized water as a negative control.

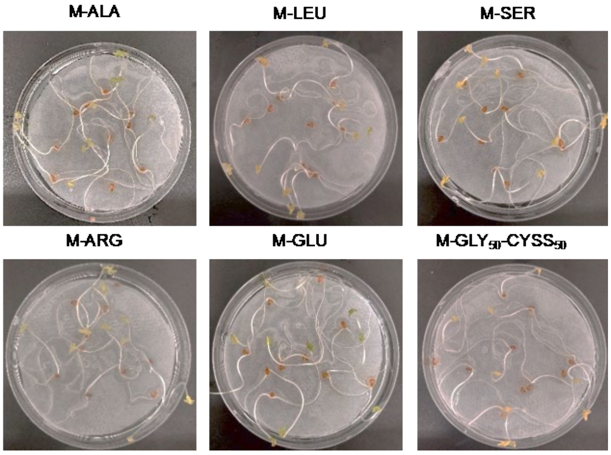

Figure S15. *Lepidium sativum* seedling growth after seed exposure to 0.156 mg mL^{-1} PAA solutions after an incubation time of 120 h.

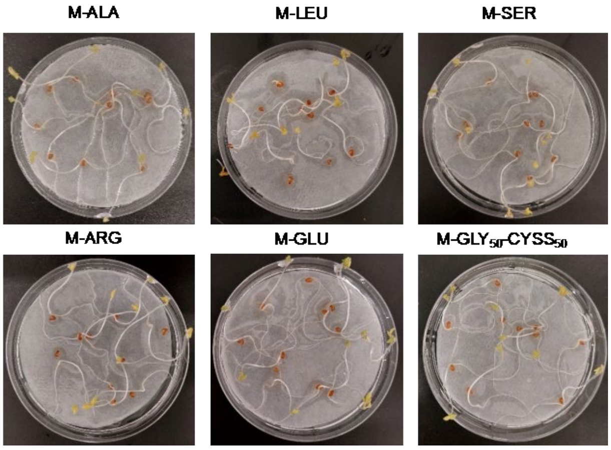

Figure S16. *Lepidium sativum* seedling growth after seed exposure to 0.313 mg mL^{-1} PAA solutions after an incubation time of 120 h.

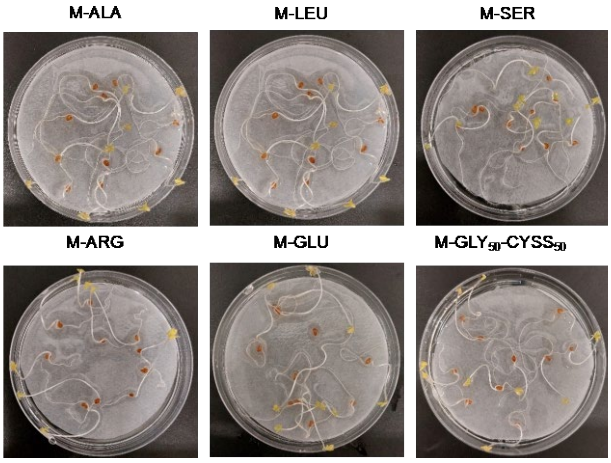

Figure S17. *Lepidium sativum* seedling growth after seed exposure to 0.625 mg mL^{-1} PAA solutions after an incubation time of 120 h.

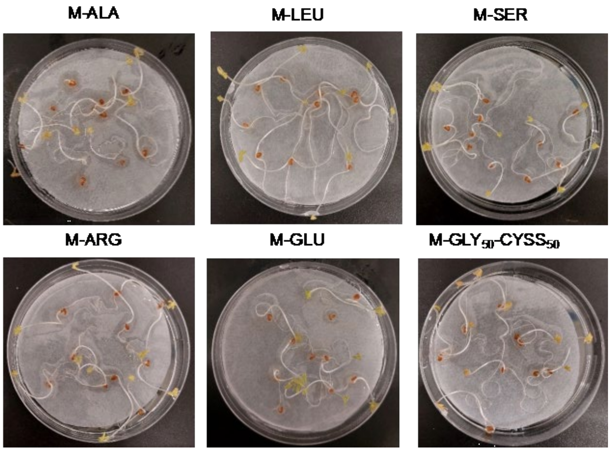

Figure S18. *Lepidium sativum* seedling growth after seed exposure to 1.25 mg mL^{-1} PAA solutions after an incubation time of 120 h.

Table S1. Percent germination of *Lepidium sativum* seeds exposed to PAA water solutions at different concentrations after an incubation time of 120 h.

PAA concentration ($mg\ mL^{-1}$)	SG% [a]					
	M-ALA	M-LEU	M-SER	M-ARG	M-GLU	M-GLY_{50}-$CYSS_{50}$
0.125	100	95	100	100	98	100
0.313	100	98	100	100	100	100
0.625	100	98	98	98	100	98
1.25	100	100	95	98	95	100
2.5	95	100	100	100	100	98

[a] Seed germination percentage calculated following Eq. 1.

Table S2. Relative seed germination of *Lepidium sativum* seeds exposed to PAA water solutions at different concentrations after an incubation time of 120 h.

PAA concentration ($mg\ mL^{-1}$)	RSG [a]					
	M-ALA	M-LEU	M-SER	M-ARG	M-GLU	M-GLY_{50}-$CYSS_{50}$
0.125	1.00	0.97	1.00	1.00	1.00	1.03
0.313	1.03	1.00	1.00	1.03	1.05	1.05
0.625	1.00	1.00	1.00	0.98	1.00	1.03
1.25	1.00	1.05	0.95	0.98	0.95	1.00
2.5	0.97	1.03	1.05	1.00	1.00	0.98

[a] Relative seed germination calculated following Eq. 2.

Table S3. Relative Radicle Growth of *Lepidium sativum* seedlings in PAA water solutions at different concentrations after an incubation time of 120 h.

PAA concentration (mg mL^{-1})	RRG [a]					
	M-ALA	M-LEU	M-SER	M-ARG	M-GLU	M-GLY$_{50}$-CYSS$_{50}$
0.125	1.22 ± 0.51	1.04 ± 0.35	0.92 ± 0.16	0.77 ± 0.26	0.93 ± 0.24	1.16 ± 0.29
0.313	1.02 ± 0.30	0.94 ± 0.30	1.15 ± 0.29	0.66 ± 0.18	0.99 ± 0.20	0.98 ± 0.41
0.625	0.93 ± 0.22	0.77 ± 0.20	1.07 ± 0.31	0.54 ± 0.15	0.80 ± 0.21	0.82 ± 0.21
1.25	0.68 ± 0.21	0.75 ± 0.18	1.08 ± 0.27	0.52 ± 0.16	0.82 ± 0.28	0.59 ± 0.15
2.5	0.24 ± 0.08	0.49 ± 0.18	0.89 ± 0.25	0.32 ± 0.09	0.87 ± 0.42	0.37 ± 0.12

[a] Relative radicle growth calculated following Eq. 3.

Table S4. Germination index of *Lepidium sativum* seeds exposed to PAA water solutions at different concentrations after an incubation time of 120 h.

PAA concentration (mg mL^{-1})	GI [a]					
	M-ALA	M-LEU	M-SER	M-ARG	M-GLU	M-GLY$_{50}$-CYSS$_{50}$
0.125	1.22 ± 0.51	1.01 ± 0.34	0.92 ± 0.16	0.77 ± 0.26	0.93 ± 0.24	1.20 ± 0.30
0.313	1.04 ± 0.30	0.94 ± 0.30	1.15 ± 0.29	0.67 ± 0.19	1.04 ± 0.21	1.03 ± 0.43
0.625	0.93 ± 0.22	0.77 ± 0.20	1.07 ± 0.31	0.53 ± 0.15	0.80 ± 0.21	0.84 ± 0.21
1.25	0.68 ± 0.21	0.79 ± 0.19	1.03 ± 0.26	0.50 ± 0.16	0.78 ± 0.26	0.59 ± 0.15
2.5	0.23 ± 0.08	0.50 ± 0.18	0.93 ± 0.26	0.32 ± 0.09	0.87 ± 0.42	0.36 ± 0.11

[a] Germination index calculated following Eq. 4.

References

[1] F. Lazzari, A. Manfredi, J. Alongi, R. Mendichi, F. Ganazzoli, G. Raffaini, P. Ferruti, E. Ranucci, Self-structuring in water of polyamidoamino acids with hydrophobic side chains deriving from natural α-amino acids, Polymers 10(11) (2018) https://doi.org/10.3390/polym10111261

[2] P. Ferruti, N. Mauro, L. Falciola, V. Pifferi, C. Bartoli, M. Gazzarri, F. Chiellini, E. Ranucci, Amphoteric, prevailingly cationic l-arginine polymers of poly(amidoamino acid) structure: synthesis, acid/base properties and preliminary cytocompatibility and cell-permeating characterizations, Macromol. Biosci. 14(3) (2014) 390-400, https://doi.org/10.1002/mabi.201300387

[3] A. Beduini, F. Carosio, P. Ferruti, E. Ranucci, J. Alongi, Synergism between α-amino acid-derived polyamidoamines and sodium montmorillonite for enhancing the flame retardancy of cotton fabrics, Polym. Degrad. Stabil. 225 (2024) 110764, https://doi.org/10.1016/j.polymdegradstab.2024.110764

[4] Y. Luo, J. Liang, G. Zeng, M. Chen, D. Mo, G. Li, D. Zhang, Seed germination test for toxicity evaluation of compost: its roles, problems and prospects, Waste Manag. 71 (2018) 109-114, https://doi.org/10.1016/j.wasman.2017.09.023
